# Targeted proteoform mapping uncovers specific Neurexin-3 variants required for dendritic inhibition

**DOI:** 10.1016/j.neuron.2022.04.017

**Published:** 2022-07-06

**Authors:** David Hauser, Katharina Behr, Kohtarou Konno, Dietmar Schreiner, Alexander Schmidt, Masahiko Watanabe, Josef Bischofberger, Peter Scheiffele

**Affiliations:** 1 Biozentrum of the University of Basel, Spitalstrasse 41, 4056 Basel, Switzerland; 2 Department of Biomedicine, University of Basel, Pestalozzistrasse 20, 4056 Basel, Switzerland; 3 Department of Anatomy, Faculty of Medicine, Hokkaido University, Sapporo, Japan

**Keywords:** interneuron, alternative splicing, proteoform, targeted proteomics, synaptic specificity, synaptic adhesion, RNA, neuronal circuit, GABA, autism

## Abstract

The diversification of cell adhesion molecules by alternative splicing is proposed to underlie molecular codes for neuronal wiring. Transcriptomic approaches mapped detailed cell-type-specific mRNA splicing programs. However, it has been hard to probe the synapse-specific localization and function of the resulting protein splice isoforms, or “proteoforms,” *in vivo*. We here apply a proteoform-centric workflow in mice to test the synapse-specific functions of the splice isoforms of the synaptic adhesion molecule Neurexin-3 (NRXN3). We uncover a major proteoform, NRXN3 AS5, that is highly expressed in GABAergic interneurons and at dendrite-targeting GABAergic terminals. NRXN3 AS5 abundance significantly diverges from *Nrxn3* mRNA distribution and is gated by translation-repressive elements. *Nrxn3 AS5* isoform deletion results in a selective impairment of dendrite-targeting interneuron synapses in the dentate gyrus without affecting somatic inhibition or glutamatergic perforant-path synapses. This work establishes cell- and synapse-specific functions of a specific neurexin proteoform and highlights the importance of alternative splicing regulation for synapse specification.

## Highlights

• Translational regulation guides alternative Neurexin proteoform expression
• NRXN3 AS5 proteoforms are concentrated at dendrite-targeting interneuron synapses
• A proteome-centric workflow uncovers NRXN3 AS5 interactors *in vivo*
• Loss of NRXN3 AS5 leads to selective impairments in dendritic inhibition

## Teaser

Hauser et al. use the genetic tagging of a single alternative exon, targeted proteomics, and loss-of-function models to analyze the synaptic localization and function of splice isoforms of synaptic adhesion molecules. They demonstrate that post-transcriptional control generates specific splice isoforms for dendrite-targeting GABAergic synapses.

## Introduction

The formation and functional specification of synapses are fundamental for neuronal circuit operation. During development, molecular programs shape synapse formation and function (Chowdhury et al., 2021; de Wit and Ghosh, 2016; Favuzzi and Rico, 2018; Gomez et al., 2021; Jang et al., 2017; Paul et al., 2017; Sanes and Yamagata, 2009; Yuzaki, 2018). Terminal gene batteries direct the cell-type-specific expression of key molecular constituents that encode components of neurotransmitter synthesis, release, neurotransmitter receptors, and synaptic adhesion molecules (Hobert, 2016). Major innovations in transcriptomic and proteomic approaches have advanced our understanding of cell-type- and synapse-specific molecular repertoires that contribute to the specification of synaptic connectivity and function (Apóstolo et al., 2020; Favuzzi et al., 2019; Kurmangaliyev et al., 2020; Mayer et al., 2018; Mi et al., 2018; Paul et al., 2017; Ribeiro et al., 2019; Sanes and Zipursky, 2020; Savas et al., 2015; Schreiner et al., 2017; Takano et al., 2020). Recent work highlighted the extensive modification of neuronal wiring regulators at the level of alternative mRNA splicing, producing distinct cellular transcript isoform repertoires (Furlanis et al., 2019; Matsuda et al., 2016; Ray et al., 2020; Saito et al., 2019; Traunmüller et al., 2016; Vuong et al., 2018; Wamsley et al., 2018; Zheng et al., 2012). Evolutionary comparisons of alternative splicing across species uncovered a massive expansion of alternative exon usage from invertebrates to mammals, non-human primates, and humans (Barbosa-Morais et al., 2012; Merkin et al., 2012). Thus, increased molecular diversification by alternative splicing was proposed to be a major driver of phenotypic diversity. Consistent with this hypothesis, manipulation of individual alternative exons in single genes results in specific functional and structural synaptic deficits (Aoto et al., 2013; Feng et al., 2021; Miura et al., 2013; Nguyen et al., 2016; Park et al., 2020; Quesnel-Vallières et al., 2015; Thalhammer et al., 2017; Uchigashima et al., 2020; Um et al., 2016; Yap et al., 2016). Thus, alternative splice variants of synaptic proteins are thought to underlie a cell- and synapse-specific code for neuronal wiring (Furlanis and Scheiffele, 2018; Gomez et al., 2021; Takahashi and Craig, 2013).

Although methodologies for deep profiling of transcript isoforms continue to rapidly advance, there are major limitations in probing to what extent such transcript isoforms contribute to functionally relevant protein diversity *in vivo*. Quantitative transcriptome—proteome correlations—led to the conclusion that only 40% of protein level variance can be explained by mRNA levels (Aebersold et al., 2018; Liu et al., 2016; Smith et al., 2013; Vogel and Marcotte, 2012). Noncoding 5′ and 3′ untranslated regions are major regulators of translation, protein localization, and protein-protein interactions (Mayr, 2017). Such post-transcriptional regulation is particularly prevalent in the nervous system (Holt et al., 2019; Tushev et al., 2018). Moreover, subcellular localization of splice variants—which is central for neuronal connectivity in the brain—cannot be deduced from transcriptomic analyses. Given these limitations, it remains a major question how mRNA splice isoforms contribute to functionally distinct synaptic proteoforms and a functional code for cell-type-specific synapse properties.

**Figure 1:**
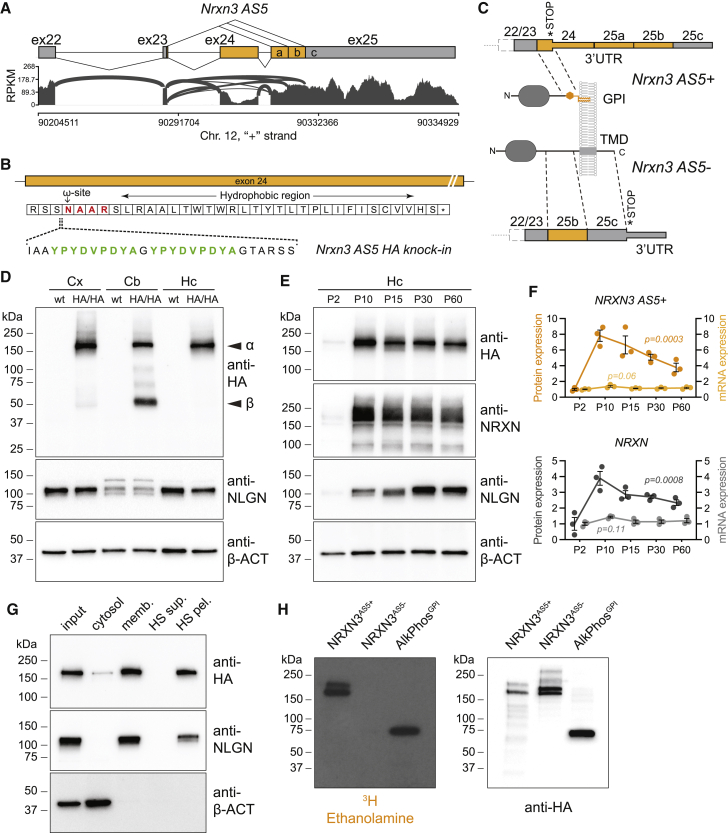
Expression and detection of NRXN3 AS5+ proteoforms in mice (A) Sashimi plots illustrating read distribution and splice junctions arising from mouse *Nrxn3 AS5* in ribosome-associated mRNAs isolated from SST interneurons in mouse hippocampus (P28). Exons are depicted as boxes, and introns as dashed lines. Alternative exons and alternative acceptor sites (a,b) are marked in orange and constitutive exons in gray. (B) Amino acids of exon 24 protein coding sequence in *Nrxn3-AS5*^*HA*^ knockin mice. The HA epitopes (green), ω-site (red), and hydrophobic stretch conferring GPI-anchoring are indicated. (C) Schematic illustrating introduction of a translational stop codon in AS5+ (exon 24-containing mRNAs). This results in production of shortened, GPI-anchored NRXN3 proteoforms encoded by mRNAs with a long 3′UTR encoded by exons 25a, 25b, and 25c. AS5- mRNA isoforms encode canonical transmembrane NRXN3 proteins. (D) Western blot of whole neocortex (Cx), cerebellum (Cb), and hippocampal (Hc) extract from P28 *wild-type* and *Nrxn3-AS5*^*HA/HA*^ knockin mice probed with anti-HA, antineuroligin (NLGN), and anti-beta-actin (β-ACT) antibodies. Position of α- and β-Neurexin proteoforms is indicated. (E) Western blot of hippocampal brain extracts across development (postnatal days 2–60) from *Nrxn3-AS5*^*HA/wt*^ knockin mice probed with anti-HA, antiNeurexin (NRXN), antineuroligin (NLGN), and anti-beta-actin (β-ACT) antibodies. (F) Quantification of protein levels for HA-tagged NRXN3-AS5+ and for PAN-NRXN across development (P2-60), and corresponding mRNA levels assessed by qPCR for exon 24 (AS5+, GPI-anchored proteoform) and exon 25 (AS5-, transmembrane proteoform) of *Nrxn3*, n = 3 animals per time point. (G) Distribution of endogenous NRXN3-AS5^HA^ protein in subcellular fractionation of hippocampal cytosolic, membrane, and high-salt (HS) washed membrane fractions (equal percentage of total sample loaded in all lanes). (H) Overexpressed HA-tagged NRXN3-AS5+, NRXN3 AS5-, and placental alkaline phosphatase proteins immunoprecipitated from transfected HEK293 cells after radiolabeling with ^3^H-ethanolamine. Immunoprecipitates were analyzed by autoradiography (left panel) or probed by western blotting with anti-HA antibodies (right panel). Mean and SEM, one-way ANOVA.

We here combined genetic tagging of an endogenous splice isoform, selective ablation, electrophysiological circuit analysis, and splice isoform-specific targeted proteomic approaches to test synapse-specific recruitment and function of splice isoforms of the synaptic adhesion molecule Neurexin-3 (NRXN3). *Nrxn3* sequence variants and mutations have been linked to alterations in emotional behavior, drug abuse, and autism (Keum et al., 2018; Vaags et al., 2012). Alternative splicing at up to six segments (AS1-6) results in the generation of thousands of *Nrxn3* mRNA isoforms in the mammalian brain (Ray et al., 2020; Schreiner et al., 2014; Treutlein et al., 2014). Although mouse knockout models for *Nrxn3* and genetic manipulation of the alternative exon at AS4 revealed some synaptic phenotypes, it is still largely unclear how splicing of *Nrxn3* affects neuronal function (Aoto et al., 2015; Aoto et al., 2013; Keum et al., 2018; Nguyen et al., 2016). Here, we focused on the *Nrxn3* AS5 segment (designated as exon 23-24-25, with exon 24 encoding the alternative exon; Figure 1A). This segment consists of multiple, evolutionarily conserved alternative splice donor and acceptor sites, and thus, it is a major contributor to *Nrxn3* mRNA isoform diversification (Schreiner et al., 2014). *Nrxn3* isoforms skip the alternative exon 24 and splice into the alternative accepter 25b encode “canonical” transmembrane proteins that interact with C1q-like proteins C1ql2,3 *in vitro* and form a ternary complex with kainate receptors at hippocampal mossy fiber synapses (Matsuda et al., 2016). However, *Nrxn3* mRNAs including exon 24 (also called AS5^+^ mRNA isoforms) do not encode transmembrane domain-containing neurexins and were proposed to be secreted (Aoto et al., 2015; Ushkaryov and Sudhof, 1993). Thus, it has remained largely enigmatic how such NRXN3 isoforms could contribute to synaptic transmission and brain function.

## Results

### Splice proteoform-specific tagging of Nrxn3 *in vivo*

mRNAs containing exon insertions at the alternatively spliced segment AS5 are widely detected in the mouse nervous system. Using Sashimi plots to visualize exon-exon junctions from deep RNA sequencing data (Furlanis et al., 2019), we observed that in mouse hippocampus, AS5^+^ mRNA isoforms mostly contain exon 23–24 and the downstream alternative acceptor side 25a (Figure 1A). By contrast, the canonical AS5^−^ variants skip exon 24 and mostly join exon 23 into the downstream acceptor sites 25b and 25c. Importantly, the alternative exon 24 contains a premature translational stop codon, and exon 24-containing *Nrxn3 AS5* mRNAs were hypothesized to be targeted by nonsense-mediated decay (Giorgi et al., 2007). However, we did not observe translation-dependent mRNA decay of endogenous *Nrxn3AS5* isoforms *in vitro* (Figure S1A). This raises the question whether AS5^+^ proteoforms are expressed *in vivo*.

We used homology-directed genome editing with CRISPR-Cas9 and asymmetric donor DNA (Richardson et al., 2016) to directly probe NRXN3 AS5 variants on the protein level. We generated knockin mice where a double HA epitope was inserted into the coding sequence of *Nrxn3* exon 24 (Figures 1B and S1B). Heterozygous and homozygous *Nrxn3AS5*^*HA*^ mice were viable and fertile and did not show visible abnormalities. *Nrxn3α* and *Nrxn3β* mRNA levels and the overall proteome were not significantly altered in *Nrxn3AS5*^*HA*^ mice (Figures S1C and S1E). We observed a slight increase in exon 24-containing *Nrxn3* mRNAs, a small reduction in total NRXN3 protein level, animal weight, and glutamatergic and GABAergic synapse strength (mEPSC and mIPSC amplitudes) in dentate granule cells (Figures S1C–S1Η). However, frequency and decay time course of miniature synaptic currents were not affected, indicating largely normal synaptic transmission. This suggests that the knockin manipulation resulted in somewhat increased incorporation of exon 24 and a small alteration in the development of the mice (Figures S1C–S1Η). The endogenous epitope-tagged NRXN3α and NRXN3β AS5^HA^ proteins were readily detected by western blot in neocortical, hippocampal, and cerebellar tissues (Figure 1D). Although α forms were dominant in the forebrain, α and β AS5^HA^ proteoforms were significantly coexpressed in the cerebellum. Over postnatal development, NRXN3 AS5 proteoforms increased 8-fold from postnatal day 2 (P2) to P10 and then slightly decreased until P60 (Figures 1E and 1F). Notably, the corresponding Nrxn3 AS5+ mRNA levels were unchanged over the same developmental time frame, suggesting significant post-transcriptional regulation of proteoform expression (Figure 1F).

Interestingly, the endogenous NRXN3α AS5^HA^ and NRXN3β AS5^HA^ proteins in the hippocampus were tightly associated with membrane fractions and could not be solubilized by high-salt extraction (Figure 1G). When examining the sequence of AS5^+^ variants, we observed that exon 24 encodes evolutionarily conserved amino acids that resemble GPI-anchor attachment sites (Figures 1B, 1C, and S1I). When expressed in HEK293T cells *in vitro*, NRXN3 AS5 proteins incorporated ^3^H-ethanolamine, one of the building blocks of GPI-anchors (Figure 1H). Transfer of the exon 24-encoded amino acids to a heterologous protein was sufficient to confer membrane anchoring (Figures S1J and S1K). Thus, AS5^+^ mRNAs encode noncanonical, membrane-anchored proteoforms of NRXN3.

### Cell-type-specific expression of NRXN3AS5 proteoforms

**Figure 2:**
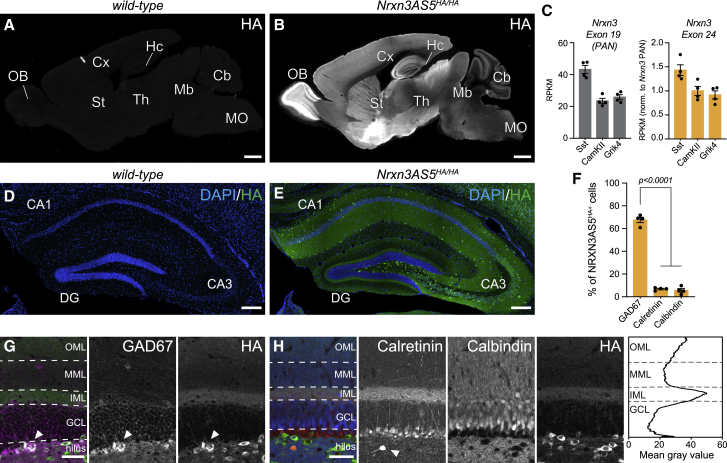
Selective expression of NRXN3 AS5+ proteoforms (A and B) Detection of NRXN3-AS5^HA^ proteins in 28 day old *wild-type* (A) and homozygous *Nrxn3-AS5*^*HA*^ mice (B); OB, olfactory bulb, Cx, cortex, St, striatum, Hc, hippocampus, Th, thalamus, Mb, midbrain, Cb, cerebellum, and MO, medulla oblongata. (C) RNA sequencing reads for constitutive (exon 19) and alternative exon 24 of *Nrxn3*, from ribosome-associated mRNAs isolated from SST^cre^, CamKII^cre^ (CA1), and Grik4^cre^ (CA3)—defined hippocampal cell populations. Read counts extracted from published data (Furlanis et al., 2019). (D and E) Detection of NRXN3-AS5^HA^ proteins in hippocampus of *wild-type* (D) and *Nrxn3-AS5*^*HA/HA*^ mice (E). (F) Fraction of NRXN3-AS5^HA^ protein expressing cells in the hilus of dentate gyrus coexpressing GAD67, calbindin (CB), or calretinin (CR), N = 4 mice, n = 3–4 brain slices per mouse, P25-30. (G and H) Colocalization analysis of NRXN3-AS5^HA^ protein (HA, green) with (G) GAD67 (magenta), arrowheads indicate colocalized example cell and (H) calretinin (CR, red) and calbindin (CB, blue) in dentate gyrus in *Nrxn3-AS5*^*HA/HA*^ knockin mice. Note that calretinin is expressed in glutamatergic mossy cells as well as (at higher level) in Cajal-Retzius cells (indicated with arrow head in [H]). Right panel in (H) shows a line scan of HA staining intensity across layers of dentate gyrus (Average of N = 3 animals, n = 2 ROIs per animal). OML, outer molecular layer, MML, middle molecular layer, IML, inner molecular layer, and GCL, granule cell layer. Mean and SEM, two-way ANOVA followed by Bonferroni’s test. Scale bars, 1 mm in (A and B); 200 μm in (D and E); and 50 μm in (G and H).

HA-tagged proteins were widely and selectively detected in the brain of *Nrxn3 AS5*^*HA*^ mice (Figures 2A and 2B). Given the laminar structure of the hippocampus, we used this region to examine cell- and synapse-specific expressions of NRXN3 AS5 protein and transcript isoforms in more detail. At the transcript level, AS5^+^ mRNA isoforms showed comparable expression across glutamatergic CA1 and CA3 pyramidal neurons and GABAergic somatostatin (SST) interneurons (Figure 2C), similar to previous conclusions from single-cell transcript analyses (Fuccillo et al., 2015). In contrast to the mRNA levels, the endogenous, HA-tagged AS5^+^ proteoform was almost exclusively detected in GABAergic interneurons, indicating a strong cell type selectivity at the protein level (Figures 2D–2F and S2A–S2J). In the dentate gyrus, pronounced NRXN3 AS5^HA^ immunoreactivity was concentrated in the inner molecular layer (IML), the distal outer molecular layer, and in GAD67-positive somata in the hilus (Figures 2G and 2H). By contrast, colabeling with calbindin and calretinin, markers of glutamatergic dentate granule cells and hilar mossy cells, respectively, revealed no significant NRXN3 AS5^HA^ protein expression in these cells (Figure 2H). Further analysis in area CA1 confirmed expression of NRXN3 AS5^HA^ in GABAergic cells, specifically somata of parvalbumin (PV), SST, cholecystokinin (CCK), but not nitric oxide synthase 1 (nNOS)-expressing interneuron subclasses (Figures S2A–S2I). Similarly, selective NRXN3 AS5^HA^ expression in GABAergic interneurons was detected in the molecular and granular layers of the cerebellum (Figure S2J). Thus, the tagging of an endogenous NRXN3 splice variant uncovers an unexpected cell type selectivity of the corresponding proteoform.

**Figure 3:**
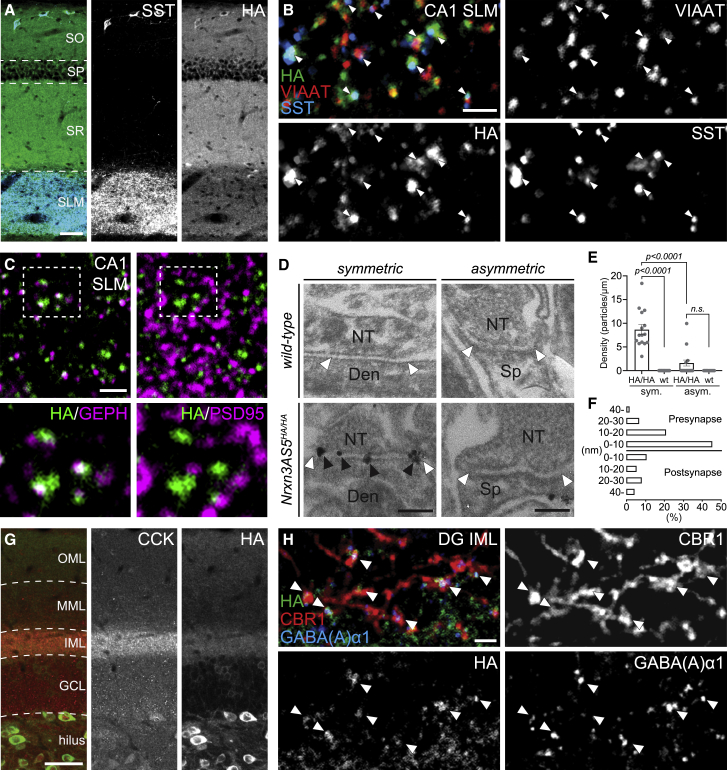
Presynaptic localization of NRXN3 AS5+ isoforms (A) Overview of NRXN3-AS5^HA^ protein (HA, green) and somatostatin (SST, blue) labeling in hippocampal CA1, SO, stratum oriens, SP, stratum pyramidale, SR, stratum radiatum, and SLM, stratum lacunosum moleculare. (B) High-magnification view of NRXN3-AS5^HA^ protein (HA, green) colocalization with VIAAT (red) and somatostatin (SST, blue) in CA1 SLM. (C) High-magnification view of NRXN3-AS5^HA^ protein (HA, green) colocalization with gephyrin (GEPH, magenta) and PSD-95 (magenta) in CA1 SLM, lower panels magnified view of indicated area. (D) Pre-embedding immunoelectron microscopy on glyoxal-fixed tissue for NRXN3-AS5^HA^ protein localization at symmetric and asymmetric synapses in SLM of wild-type control and *Nrxn3 AS5*^*HA/HA*^ mice. The edge of the postsynaptic specializations at asymmetrical and symmetrical synapses are each indicated by a pair of white arrowheads. Each immunogold particle is indicated by a black arrowhead, NT, nerve terminal, Den, dendrite, Sp, spine. For overview images, see Figure S3. (E) The density of metal particles detected per 1 μm of synaptic cleft calculated from 15 symmetric and 20 asymmetric synapses from n = 4 sections and N = 2 *Nrxn3AS*^*HA/HA*^ mice, and 17 symmetric and 18 asymmetric synapses from n = 4 sections and N = 2 wild-type mice. Note that there is no statistically significant difference in labeling density of asymmetric synapses as compared with wild-type mice. (F) Vertical distribution of 78 particles from the midline of synaptic cleft across 15 symmetric synapses from n = 4 sections and N = 2 *Nrxn3AS*^*HA/HA*^ mice. (G) Overview of NRXN3-AS5^HA^ protein (HA, green) and cholecystokinin (CCK, red) labeling in the hilus of the dentate gyrus, OML, outer molecular layer, MML, middle molecular layer, IML, inner molecular layer, and GCL, granule cell layer. (H) High-magnification view of NRXN3-AS5^HA^ protein (HA, green) colocalization with cannabinoid receptor 1 (CBR1, red) and GABA_A_-receptor subunit alpha 1 (GABA_A_α1, blue) in dentate gyrus IML. Scale bars, 50 μm in (A and G); 2 μm in (B, C, and H); and 200 nm in (D). Note that all experiments (except G) were performed on glyoxal-fixed tissue. Mean and SEM, one-way ANOVA followed by Tukey’s multiple comparison.

Although NRXN3 AS5^HA^ detection in perinuclear compartments demonstrated its interneuron restriction, higher magnification analysis with protocols that optimize detection of synaptic antigens uncovered a concentration at GABAergic synapses (Figure 3; see STAR Methods for details). In *stratum lacunosum moleculare* (SLM) of CA1, an area rich in GABAergic synapses formed by SST interneurons (Figures 3A and S2K), NRXN3 AS5^HA^ concentrates in structures containing SST and vesicular inhibitory amino acid transporter VIAAT (Figure 3B) and is apposed to the GABAergic postsynaptic marker Gephyrin (Figure 3C). By contrast, no apposition was observed for the glutamatergic postsynaptic marker PSD95 (Figure 3C). Pre-embedding immunoelectron microscopy in CA1 SLM showed significant labeling at symmetric synapses on dendritic shafts, but not asymmetric synapses on spines (Figures 3D, 3E, and S3A). The vertical distribution from the midline of the synaptic cleft to the center of metal particles peaked in a presynaptic 0–10-nm bin, with the mean distance of 13.4 ± 1.7 nm presynaptic from the midline of the synaptic cleft (Figure 3F). Considering the size of antibodies and gold particles, this biased distribution suggests an exclusive presynaptic localization of NRXN3 AS5^HA^. In cultured hippocampal neurons, punctate NRXN3 AS5^HA^ labeling is closely colocalized with GABAergic but not glutamatergic synapse markers (Figures S3B–S3F). Moreover, in the dentate gyrus, NRXN3 AS5^HA^ was highly concentrated in the IML (Figure 3G), where axons of cholecystokinin (CCK)/cannabinoid receptor 1 (CB1R)-expressing interneurons (also referred to as “hilar commissural and association pathway projecting cells,” HICAP cells) terminate on proximal dendrites of granule cells (Halasy and Somogyi, 1993; Hefft and Jonas, 2005; Hosp et al., 2014). Here, NRXN3 AS5^HA^ colocalized with both CCK and CBR1 and was apposed to the postsynaptic GABA_A_-receptor α1 subunit (Figures 3G and 3H). By comparison, only little NRXN3 AS5^HA^ immunoreactivity was detected at synaptotagmin-2 (SYT2)-positive perisomatic sites in the dentate granule cell layer (Figure S3G) that represent parvalbumin-interneuron synapses (Sommeijer and Levelt, 2012). Thus, the selective tagging of the endogenous NRXN3 AS5 proteoform uncovered an unexpected localization at specific populations of GABAergic synapses *in vivo*.

### Isolation of native NRXN3AS5 protein complexes

**Figure 4:**
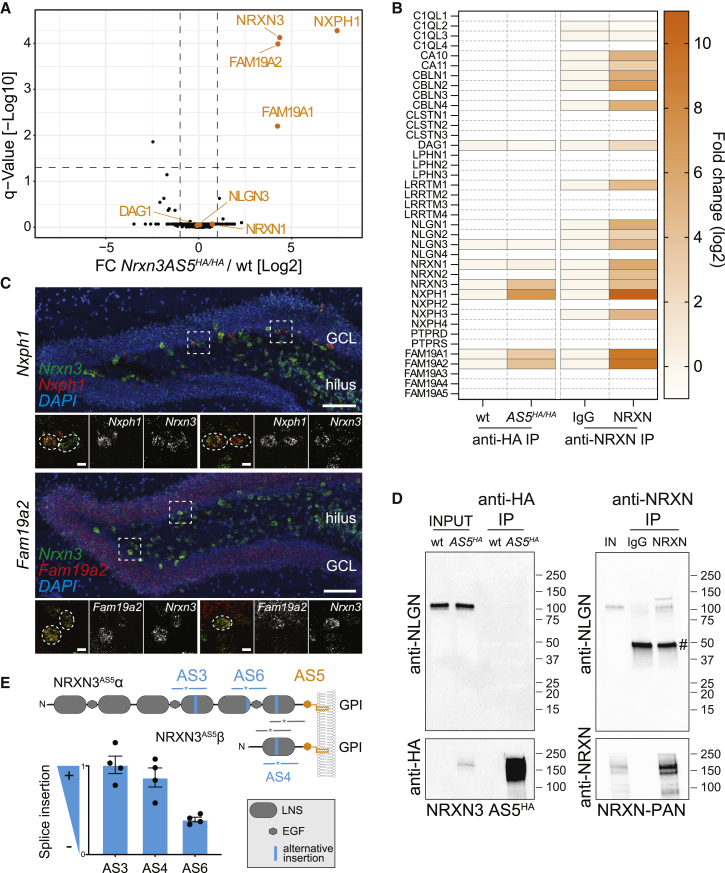
NRXN3 AS5+ proteoforms recruit specific synaptic ligands (A) Volcano plot of protein abundance (iBAQ, log_2_ scale fold change knockin versus WT mice and q value) in anti-HA immunoprecipitates from hippocampi from *Nrxn3-AS5*^*HA/HA*^ and *wild-type* (negative control) P25-30 mice (N = 5 mice per genotype). Selected known Neurexin ligands detected in the analysis are marked in orange. See Table S1 for detailed data. (B) Heatmap of protein abundance (iBAQ, log_2_ scale) of known Neurexin ligands recovered from *wild-type* and *Nrxn3-AS5*^*HA/HA*^ mice in anti-HA immunoprecipitates and recovered from wild-type mice in control IgG and anti-NRXN immunoprecipitates, respectively. The anti-NRXN antibody is raised against the cytoplasmic tail of NRXN1 nd cross-reacts with all transmembrane NRXNs (Muhammad et al., 2015). *Nrxn3 AS5*^*HA/HA*^ versus *wild type*: q < 0.001 for NRXN3, NXPH1, FAM19A1, and FAM19A2; q > 0.05 for NRXN1, NLGN3, and DAG1. Anti-NRXN versus control IgG: q < 0.001 for all except C1QL2 (q = 0.0066) and C1QL3 (q = 0.08) (multiple t test with Benjamini, Krieger, and Yekutieli correction). See Table S2 for NRXN immunoprecipitates, and Table S3 for details on selected ligands. Nondetectable proteins depicted as boxes with dashed outline. (C) Fluorescent *in situ* hybridization for *Nrxn3* and ligands *Nxph1* and *Fam19A2* transcripts in dentate gyrus of P30 mice. (D) Confirmation of differential ligand interactions by western blotting. Input (IN, 1%) and immunoprecipitates with anti-HA (left) or control IgG and anti-NRXN1 antibodies (right) probed with anti-NLGN (top) and anti-HA or anti-NRXN antibodies (bottom). Molecular weight markers indicated in kDa. # indicates heavy IgG-chains. (E) Schematic of NRXN3 domain organization, alternatively spliced segments (blue), and proteotypic peptides (PTPs) of constitutive/common (gray) and proteoform-specific (blue) amino acids indicated, which are quantified for AS3, AS4, and AS6, normalized to recombinant protein expressing all splice isoforms (100% splice site inclusion) and to constitutive exons. Mean and SEM, Scale bars, 100 μm (overview) and 10 μm (high-magnification images).

Neurexin proteins interact with an array of extracellular ligands. However, in vitro binding/affinity chromatography approaches do not allow for a targeted isolation of binding partners associating with the protein *in vivo*. We took advantage of the tagged endogenous NRXN3 AS5^HA^ proteoform for affinity isolation of native neurexin-ligand complexes from mouse hippocampus. Shotgun mass-spectrometry of anti-HA immunoprecipitates from detergent-solubilized hippocampus of *Nrxn3 AS5*^*HA/HA*^ mice and comparison to negative control precipitates from wild-type mice identified 3 proteins as major endogenous NRXN3 AS5 interactors (Figure 4A; *Nrxn3AS5*^*HA/HA*^ versus *wild-type* fold−change > 2.0 and q < 0.05; Table S1): FAM19A1, FAM19A2, and Neurexophilin-1 (NXPH1), three proteins previously identified as Neurexin ligands of canonical transmembrane neurexins (Born et al., 2014; Khalaj et al., 2020; Missler and Sudhof, 1998). We used cellular assays and confirmed interaction of all three ligands with the NRXN3 AS5 proteoform in heterologous cells (Figures S4A and S4B, note that transmembrane and NRXN3 AS5 proteoforms show similar binding to the three ligands). We then explored whether the Neurexin ligand mRNAs are expressed in the NRXN3 AS5-containing interneurons in the dentate gyrus. We observed high *Nxph1* and *Fam19a2* mRNA expression in *Nrxn3*-positive neurons of the hilus (Figure 4C). In addition, *Fam19a2* is broadly expressed in the granule cell layer. Considering that FAM19A1 and A2 were shown to associate with Neurexins in the biosynthetic pathway (Khalaj et al., 2020), we hypothesize that both NXPH1 and FAM19A1/A2 proteins interact with NRXN3 AS5 proteins in *cis*.

Notably, the native NRXN3 AS5 complexes lacked an array of other Neurexin ligands that were recovered by affinity isolation with antibodies to transmembrane NRXN proteins under the same experimental conditions. Thus, Neuroligins and LRRTMs, two major classes of neurexin ligands (de Wit et al., 2009; Linhoff et al., 2009; Südhof, 2017), were not recovered as hippocampal NRXN3 AS5 interactors by shotgun mass-spectrometry (Figure 4B) or western blotting (Figure 4D; Tables S2 and S3). We hypothesized that this selective recruitment of interactors by the native NRXN3 AS5 protein might result from splice insertions at additional alternatively spliced segments that gate ligand interactions. To test this, we developed and optimized splice isoform-specific targeted parallel reaction monitoring (PRM) assays (Maiolica et al., 2012; Schreiner et al., 2015). Conventional shotgun proteomics stochastically samples a random portion of the proteome. By contrast, PRM assays use optimized separation and detection for a subset of preselected proteotypic peptides (PTPs) that are specific to a protein or proteoform of interest. PTPs are detected based on their chromatographic retention time and mass to charge ratio of preselected fragments (transitions) with an isotopically labeled reference peptide serving as an internal standard for quantification. The NRXN3 AS5 proteins immunoprecipitated from mouse hippocampal tissue contained almost exclusively alternative insertions at AS3 and AS4 with intermediate incorporation of insertions at AS6 (Figures 4E, S4C, and S4D). Notably, AS4 insertions significantly reduce affinity for interaction with neuroligins and LRRTMs (Siddiqui et al., 2010), thus providing a potential mechanism for the observed ligand selectivity of native NRXN3 AS5 proteins. In sum, this analysis uncovers a selective synaptic splice code for hippocampal GABAergic neurons at the protein level.

### Deletion of alternative exon 24 results in the loss of NRXN3 AS5 proteins

**Figure 5:**
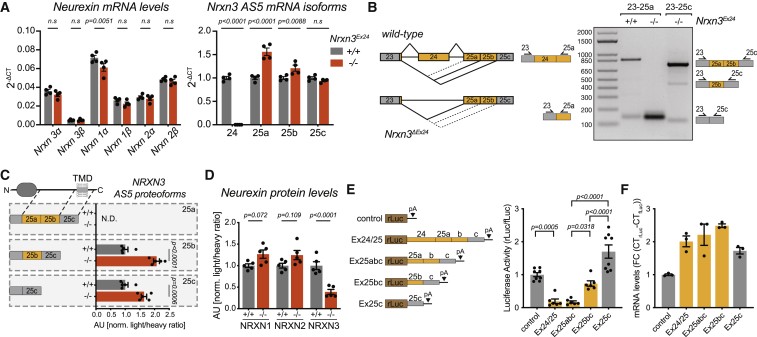
Translational silencing gates NRXN3 protein expression (A) Quantitative PCRs of major *Nrxn* transcript isoforms (left panel) and mRNAs containing exon 24, alternative accepters 25a, 25b, and the constitutive exon 25c (right panel, plotted relative to *wild type*) for *wild type* and *Nrxn3*^*ΔEx24*^ mice, normalized to *Gapdh*, hippocampus, P25-30, N = 4 mice per genotype. (B) Schematic diagram illustrating alternative splicing events in *wild-type* and *Nrxn3*^*ΔEx24*^ hippocampus (left) and semiquantitative PCR visualizing *Nrxn3* transcript variants arising from alternative splicing at AS5 in *wild-type* and *Nrxn3*^*ΔEx24*^ mice (right). Position of primer binding sites on alternative exon segments is illustrated. See STAR Methods for details. (C and D) Detection of AS5 proteoforms by targeted proteomics with heavy peptides targeting alternative accepters 25a, 25b, and constitutive exon 25c (C) or targeting all NRXN1, NRXN2, and NRXN3 proteoforms (D). Ratios of light to heavy peptide detection are displayed in reference to wild-type samples of each peptide. One representative peptide shown, consistent results were obtained for multiple proteotypic peptides for the same proteoform (see Table S6), hippocampus, P25-20, N = 5 mice per genotype. (E and F) Luciferase assay of dual-promoter plasmids expressing firefly (fLuc) and renilla (rLuc) luciferase, left panel: schematic representation of different *Nrxn3* exonic sequences fused after the translational stop codon of rLuc, right panel: luciferase activity from renilla luciferase constructs normalized to firefly luciferase activity, N = 2–3 cell cultures, n = 2–3 replicates per culture (E) and mRNA levels determined by RT-qPCR of renilla luciferase constructs normalized to firefly luciferase, N = 3 cell cultures (F) (For sequences of luciferase constructs see Table S4). Mean and SEM, with two-way or one-way ANOVA followed by Bonferroni’s test for both qPCR and proteomic analysis or luciferase assay, respectively.

To explore the functional relevance of NRXN3 AS5 proteoforms, we generated AS5 knockout mice by CRISPR-Cas9-mediated genome editing with two guide RNAs targeting sequences flanking exon 24. Nonhomologous end joining resulted in a 1,309-bp deletion that removed the entire alternative exon 24 (*Nrxn3*^*ΔEx24*^ mice; Figures S5A and S5B). Heterozygous and homozygous *Nrxn3*^*ΔEx24*^ mice were born at Mendelian frequencies, were fertile, but exhibited significantly reduced weight (Figure S5C). The mRNA levels of the primary Neurexin transcripts (*Nrxn1,2,3* α and β) were unchanged in the hippocampus of *Nrxn3*^*ΔEx24*^ mice (Figure 5A). Given the presence of multiple downstream acceptor sites in exon 25 (25a, 25b, and 25c) (Schreiner et al., 2014), we examined which of these sites would be incorporated in the cells formerly including exon 24 at AS5. Quantitative PCR confirmed the complete loss of exon 24 and uncovered a significant increase in exon 25a, whereas (the constitutive) exon 25c was unaltered (Figure 5A). Thus, in the absence of exon 24, cells that previously produced AS5+ isoforms now produce mRNAs containing exon 25a (Figure 5B). This interpretation was further supported by semiquantitative PCR with oligonucleotide primers flanking AS5 that identified abundant exon 23-, exon 24-, exon 25a-containing mRNA isoforms in *wild type* and exon 23–25a containing mRNA isoforms in *Nrxn3*^*ΔEx24*^ hippocampus, respectively (Figure 5B; note that the length of the amplicon precludes quantitative detection of mRNAs with primers exon 23–25c from *wild-type* hippocampus). We then applied targeted proteomics (PRM) to directly quantify Neurexin proteoforms in the mutant mice. Interestingly, the peptides encoded by exon 25a were not detectable in *wild-type* or *Nrxn3*^*ΔEx24*^ mice, despite sensitive detection of recombinant proteins in the same assay (Figures 5C and S5D). At the same time, the canonical transmembrane NRXN3 proteoforms (detected based on 25b- and 25c-encoded peptide sequences) were elevated (Figure 5C). We speculated that cells that produce the NRXN3 AS5 proteoform in wild type do not convert upon exon 24 knockout to the canonical NRXN3 transmembrane forms but rather no protein at all, essentially representing a NRXN3 AS5 protein knockout. Consistent with this idea, the total NRXN3 protein level (assessed with pan-NRXN3 PRM assays detecting α and β isoforms) was reduced by 61 ± 3 % in the hippocampus of *Nrxn3*^*ΔEx24*^ mice (Figure 5D; similar observations made in neocortex and cerebellum; Figure S5E). We hypothesized that exon 25a might confer translational silencing of *Nrxn3* mRNAs. To test this hypothesis, we examined translational output from luciferase reporters containing as 3′UTR the various exon 25 sequences including or lacking 25a in Neuro2A cells (Figure 5E). Indeed, we observed a strong exon 25a-dependent repression of mRNA translation (Figures 5E and 5F; no alteration in mRNA abundance for the reporters of various *Nrxn3* isoforms). Collectively, these data suggest that in *Nrxn3*^*ΔEx24*^ mice NRXN3 AS5 protein is lost, whereas the canonical transmembrane NRXN3 proteoform is slightly elevated. Thus, *Nrxn3*^*ΔEx24*^ mice provide a unique opportunity to directly test the function of this noncanonical NRXN3 AS5 isoform under conditions where canonical transmembrane NRXN3 is intact.

### Loss of NRXN3 AS5 results in impaired synaptic transmission at dendrite-targeting interneuron synapses

**Figure 6:**
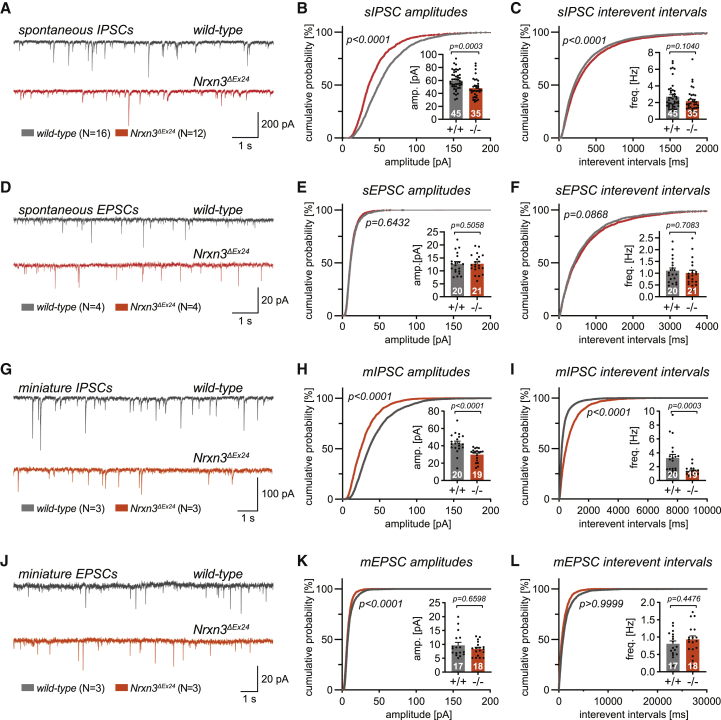
Impaired GABAergic synaptic transmission in *Nrxn3*^*ΔEx24*^ mice (A) Representative traces of spontaneous IPSCs from 6- to 8-week-old *wild-type* and homozygous *Nrxn3*^*ΔEx24*^ mice recorded in the presence of AP5 and NBQX. (B and C) Cumulative frequency distributions of amplitudes (B) and interevent intervals (C) of dentate gyrus granule cell sIPSCs recorded from *wild-type* (N = 16 animals, n = 45 cells) and homozygous *Nrxn3*^*ΔEx24*^ mice (N = 12 animals, n = 35 cells). Insets: average sIPSC amplitudes (B) and frequencies (C) per cell. (D) Representative traces of spontaneous EPSCs from 6- to 8-week-old *wild-type* and homozygous *Nrxn3*^*ΔEx24*^ mice recorded in the presence of Picrotoxin. (E and F) Cumulative frequency distributions of amplitudes (E) and interevent intervals (F) of dentate gyrus granule cell sEPSCs recorded from *wild-type* (N = 4 animals, n = 20 cells) and homozygous *Nrxn3*^*ΔEx24*^ mice (N = 4 animals, n = 21 cells). Insets: average sEPSC amplitudes (E) and frequencies (F) per cell. (G) Representative traces of miniature IPSCs from 6- to 8-week-old *wild-type* and homozygous *Nrxn3*^*ΔEx24*^ mice recorded in the presence of TTX, AP5, and NBQX. (H and I) Cumulative frequency distributions of amplitudes (H) and interevent intervals (I) of dentate gyrus granule cell mIPSCs recorded from *wild-type* (N = 3 animals, n = 20 cells) and homozygous *Nrxn3*^*ΔEx24*^ mice (N = 3 animals, n = 19 cells). Insets: average mIPSC amplitudes (H) and frequencies (I) per cell. (J) Representative traces of miniature EPSCs from 6- to 8-week-old *wild-type* and homozygous *Nrxn3*^*ΔEx24*^ mice recorded in the presence of TTX and picrotoxin. (K and L) Cumulative frequency distributions of amplitudes (K) and interevent intervals (L) of dentate gyrus granule cell mEPSCs recorded from *wild-type* (N = 3 animals, n = 17 cells) and homozygous *Nrxn3*^*ΔEx24*^ mice (N = 3 animals, n = 18 cells). Insets: average mIPSC amplitudes (K) and frequencies (L) per cell. Mean and SEM, analyzed using the Mann-Whitney test.

What is the functional contribution of GPI-anchored NRXN3 AS5 isoforms to synapse formation and transmission? As our immunohistochemical analysis revealed localization of NRXN3 AS5 at dendritic GABAergic synaptic terminals, we focused on dendrite-targeting interneuron subtypes. We examined inhibitory and excitatory synaptic transmission in acute hippocampal brain slices from adult mice, focusing on dentate gyrus because of the pronounced layer-specific expression of NRXN3 AS5 (Figures 2 and 3). Recording of spontaneous inhibitory postsynaptic currents (sIPSCs) from granule cells in symmetrical Cl^−^ conditions uncovered a significant reduction in amplitudes and a shift in the distribution of sIPSCs toward larger interevent intervals in *Nrxn3*^*ΔEx24*^ mice (Figures 6A–6C; 60 ± 2 pA versus 48 ± 3 pA, p = 0.0003, n = 45 and 35 cells, N = 12–16 mice/genotype; no change in kinetics; Figure S6A). By contrast, spontaneous excitatory postsynaptic currents were unaltered (Figures 6D–6F and S6B). This was expected, given that endogenous NRXN3 AS5 protein is not detected at glutamatergic synapses in the hippocampus. Recordings of mIPSCs and mEPSCs from granule cells further confirmed a selective impairment in GABAergic transmission.

In *Nrxn3*^*ΔEx24*^ mice, the mean mIPSC frequency and amplitude were significantly reduced by about 50% (Figures 6G–6I, 3.24 ± 0.51 Hz versus 1.37 ± 0.14 Hz, p = 0.0003) and 30% (42.9 ± 2.6 pA versus 29.8 ± 1.7 pA, p < 0.0001, n = 20 and n = 19 cells, N = 3 mice/genotype), respectively. This suggests that both, the number of functional GABAergic synapses as well as its synaptic strength, are reduced in mice lacking NRXN3 AS5. Mean amplitude, frequency, and kinetics of mEPSCs were not affected (Figures 6J–6L, S6C, and S6D). These experiments uncover a selective requirement for NRXN3 AS5 proteoforms in GABAergic transmission in the dentate gyrus.

**Figure 7:**
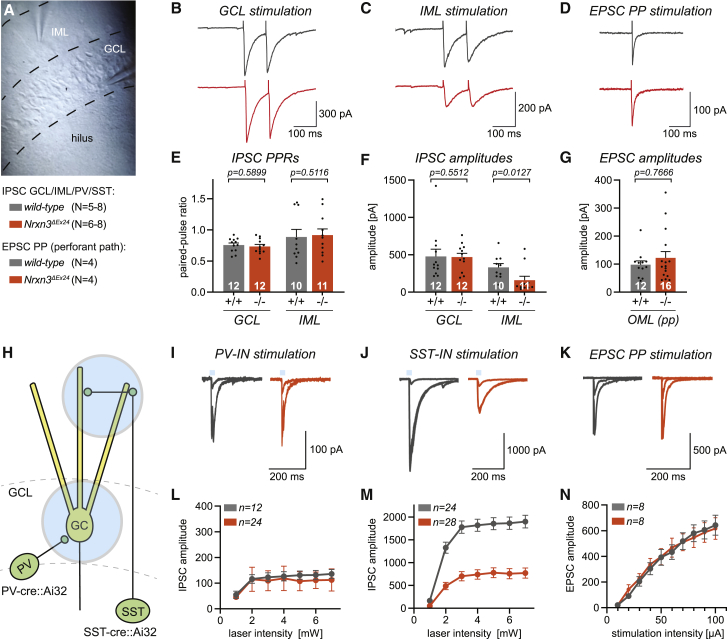
Reduced dendritic GABAergic inputs onto dentate gyrus granule cells in *Nrxn3*^*ΔEx24*^ mice (A) Positioning of electrodes to selectively stimulate GABAergic synapses in the inner molecular layer (IML) and granule cell layer (GCL) of the dentate gyrus in the presence of NBQX and AP5. (B and C) Representative traces of evoked IPSCs in response to electrical stimulation (stimulation intensity: 20 μA) in the GCL (B) and IML (C). (D) Representative traces of evoked EPSCs in response to electrical stimulation in the OML (stimulation intensity 20 μA) in the presence of picrotoxin. (E and F) Quantification of evoked IPSC paired-pulse ratios (E) and evoked IPSC amplitudes (F) upon GCL and IML stimulation recorded in dentate gyrus granule cells in *wild-type* (N = 8 animals, n = 10–12 cells) and homozygous *Nrxn3*^*ΔEx24*^ mice (N = 7–8 animals, n = 11–12 cells). (G) Quantification of evoked EPSC amplitudes upon stimulation of performant-path inputs in OML in dentate gyrus granule cells of *wild-type* (N = 4 animals, n = 12 cells) and homozygous *Nrxn3*^*ΔEx24*^ mice (N = 4 animals, n = 16 cells). (H) Schematic drawing of a dentate gyrus granule cell showing the fields of illumination in the GCL and the OML for optogenetic stimulation of inputs from PV and SST interneurons, respectively. (I and J) Representative traces of optogenetically evoked GABAergic inputs from PV (I) and SST interneurons (J). The example traces show an overlay of the evoked IPSCs to three different laser intensities (1, 2, and 4 mW) for each genotype. (K) Representative traces of glutamatergic inputs evoked by electrical perforant-path stimulation. The example traces show an overlay of the evoked EPSCs to three different stimulation intensities (10, 30, and 100 μA) for each genotype. (L) Dose-response curves showing the mean evoked IPSC amplitudes in response to optogenetic stimulation of PV interneurons recorded in dentate gyrus granule cells from *wild-type* (N = 7 animals, n = 12 cells) and homozygous *Nrxn3*^*ΔEx24*^ mice (N = 6 animals, n = 24 cells). (M) Dose-response curves showing the mean evoked IPSC amplitudes in response to optogenetic stimulation of SST interneurons recorded in dentate gyrus granule cells from *wild-type* (N = 5 animals, n = 24 cells) and homozygous *Nrxn3*^*ΔEx24*^ mice (N = 6 animals, n = 28 cells). Laser intensities ranging from 1 to 7 mW. (N) Dose-response curve showing the mean evoked EPSCs in response to electrical performant-path stimulation recorded in dentate gyrus granule cells of *wild-type* (N = 4 animals, n = 8 cells) and homozygous *Nrxn3*^*ΔEx24*^ (N = 4 animals, n = 8 cells) mice. Stimulation intensities were ranging from 10 to 100 μA. Mean and SEM, analyzed using the Mann-Whitney test.

Next, we analyzed evoked synaptic responses using selective stimulation of different interneuron subtypes, including soma-targeting PV-basket cells, as well as CCK- and SST-positive interneurons targeting proximal dendrites (IML) and distal dendrites (OML) of granule cells, respectively. GABAergic IPSCs generated by CCK-positive HICAP cells and PV-basket cells were examined by selective stimulation of axons in the IML and granule cell layer (GCL), respectively (see STAR Methods; Figures 7A–7C). Although perisomatic inhibition evoked in the GCL was unchanged, there was a significant reduction in the amplitude of postsynaptic currents evoked by IML stimulation from 332 ± 51 pA to 159 ± 54 pA (Figures 7C–7F, p = 0.0127, n = 10 and 11 cells, N = 7–8 mice/genotype). The paired-pulse ratio was comparable between genotypes consistent with a reduced number of IML synapses and/or a postsynaptic reduction of GABA_A_-receptor mediated conductance per synapse (Figure 7E). By contrast, glutamatergic transmission evoked by stimulation of perforant-path axons in the OML was unchanged in *Nrxn3*^*ΔEx24*^ mice (Figures 7D and 7G). Interestingly, immunohistochemical assessment of GABA synapses in *Nrxn3*^*ΔEx24*^ mice showed a significant reduction in the density of VIAAT/gephyrin colocalized puncta in the IML, the site of CCK-interneuron synapses, whereas the density of soma-targeting presynaptic terminals immuno-positive for the PV-interneuron marker SYT2 was unchanged (Figure S7). These observations are consistent with a selective reduction in CCK-interneuron synapse density and/or a defect in CCK synapse development.

To analyze GABAergic synapses in distal dendrites formed by SST interneurons, we transgenically expressed a cre-dependent form of channelrhodopsin [Ai32, ChR2(H134R)-EYFP::SST-Cre] in *wild-type* and *Nrxn3*^*ΔEx24*^ mice (Madisen et al., 2012). Similarly, PV-basket cells were targeted using PV-Cre::Ai32 mice. To synchronously stimulate axons of either PV- or SST-interneurons, short light pulses (2 ms) were applied to GCL or OML in PV-Cre or SST-Cre mice, respectively (Figure 7H). Systematically increasing the light intensity (1–7 mW in back focal plane) evoked saturating PV-basket cell IPSCs with similar amplitudes in *wild-type* and *Nrxn3*^*ΔEx24*^ mice (n = 12 and n = 24, respectively; Figures 7I and 7L). By contrast, the IPSC amplitude was substantially smaller in SST-interneuron synapses of *Nrxn3*^*ΔEx24*^ mice relative to wild-type animals (1,900 ± 140 pA, n = 24 versus 772 ± 110 pA, n = 28, p < 0.0001; Figures 7J and 7M). Similarly, we assessed dose-response curves of perforant-path evoked EPSCs (10–100 μA), showing that glutamatergic synaptic transmission in *wild-type* and *Nrxn3*^*ΔEx24*^ mice is not different with p > 0.5 for all intensities (Figures 7K and 7 N).

Taken together, these experiments demonstrate that the loss of NRXN3 AS5 results in a highly selective phenotype at dendrite-targeting GABAergic synapses in the dentate gyrus formed by CCK- and SST-expressing interneurons. By contrast, neither perisomatic inhibition nor glutamatergic excitatory transmission is affected.

## Discussion

The Neurexin family of adhesion molecules are critical regulators of synapse formation and function, and mutations in human *NRXN* genes predispose to neurodevelopmental disorders (Gomez et al., 2021; Siddiqui and Craig, 2011; Südhof, 2017; Yuzaki, 2018). Transcriptomic mapping supports broad expression of a large number of distinct Neurexin mRNA splice isoforms (Ray et al., 2020; Schreiner et al., 2014; Treutlein et al., 2014). However, due to technical challenges, the localization and function of the corresponding Neurexin proteoforms are not well understood. Appending epitope-tags to common (constitutive) regions of *Nrxn1* uncovered important new insights into the transport and subcellular localization of the resulting proteins (Ribeiro et al., 2019; Taniguchi et al., 2007; Trotter et al., 2019). These transmembrane Neurexins are not only detected in perinuclear membrane structures, presynaptic terminals, and axons but also in dendrites (Fairless et al., 2008; Ribeiro et al., 2019; Taniguchi et al., 2007) where (at least overexpressed) NRXN1 can inhibit neuroligins in *cis* (Taniguchi et al., 2007). In the present study, we developed quantitative, protein-centric approaches for the selective analysis of *Nrxn3 AS5* alternative splice variants *in vivo*. We find that exon 24 containing (AS5^+^) NRXN3 proteoforms are specifically expressed in GABAergic interneurons, are GPI-anchored, and are polarized to the presynaptic compartment. GPI-anchors can serve as axonal targeting signals (Dotti et al., 1991)—thus, the GPI-anchor may direct highly polarized sorting of the NRXN3 AS5 proteoforms.

Epitope-tagged endogenous NRXN3 AS5 proteoforms can be detected in the somata of PV-positive interneurons. However, we did not observe alterations in somatic inhibition in *Nrxn3*^*ΔEx24*^ mice that lack these proteoforms. This is consistent with the low detection of NRXN3 AS5 at perisomatic synapses and might be due to the significant expression of the canonical NRXN3 AS5^−^ proteins in these neurons. The NRXN3 AS5^+^ proteoforms are particularly concentrated at the dendrite-targeting GABAergic terminals *in vivo*, including CCK- and SST-interneuron synapses in the dentate gyrus. Notably, the genetic deletion of AS5^+^ isoforms selectively impairs transmission at these dendrite-targeting interneuron synapses but leaves somatic GABAergic transmission mediated by PV-interneurons and glutamatergic transmission intact. By contrast, conditional knockout of all *Nrxn3* isoforms in PV-interneurons results in a sex-specific loss of somatic GABAergic synapses in the subiculum (Boxer et al., 2021). Moreover, global genetic deletion of NRXN3 (including both, the canonical and noncanonical forms) is accompanied by broad impairments in GABAergic and glutamatergic transmission and GABAergic synapse formation (Aoto et al., 2015; Boxer et al., 2021). Thus, NRXN3 AS5^+^ proteoforms represent a specific subclass of Neurexin proteins selectively localized and required at a subpopulation of neuronal synapses.

The splice isoform-specific tag enabled us to perform an analysis of native NRXN3 AS5 protein complexes. Thus, we identified FAM19A1, FAM19A2, and neurexophilin1 (NXPH1) as a small subgroup of neurexin ligands tightly associated with the endogenous AS5^+^ NRXN3 proteoforms. The mRNAs encoding these ligands are coexpressed with *Nrxn3* in dentate gyrus interneurons, suggesting that they associate with NRXN3 AS5 in the biosynthetic pathway and might be codelivered to the presynaptic terminal. Interestingly, in cerebellar granule cells, neurexophilin-4, a protein closely related to NXPH1, selectively binds to α1- and α6-subunit containing GABA_A_-receptors. Furthermore, deletion of NXPH4 reduces GABAergic synapse number, amplitude, and frequency of mIPSCs, as well as evoked GABAergic synaptic transmission at Golgi cell to granule cell synapses, leaving glutamatergic synapses fully intact (Meng et al., 2019). Therefore, NXPH1 and NRXN3 AS5 might play a similar role in jointly orchestrating GABA_A_-receptor recruitment at distinct hippocampal inhibitory synapses. GABAergic synapses of dendrite-targeting SST-interneurons differ in many aspects from perisomatic PV-basket cell synapses including assembly and functional properties of postsynaptic GABA_A_-receptors (Lodge et al., 2021; Schulz et al., 2018). Furthermore, they were reported to show more dynamic plasticity of inhibitory synaptic transmission than PV-basket cells (Chiu et al., 2018). Therefore, GPI-anchored Neurexins might allow for a more dynamic regulation of synapse function as compared with perisomatic inhibition.

One of the major conclusions of this work is that the cell- and synapse-specific localization and function of NRXN3 AS5 cannot simply be deduced from the distribution of the *Nrxn3* mRNA. We demonstrate that the alternative *Nrxn3* exon 25a imposes powerful translational silencing and, thus, gates NRXN3 protein expression *in vivo*. Although the molecular mechanisms underlying translational silencing by exon 25a remain to be explored, it is notable that the exon 25a-encoded sequences are evolutionarily conserved. The reason for this conservation is currently unclear. One possibility would be the regulated production of small amounts of exon 25a-containing proteins upon signaling and translational derepression that would be missed in steady-state analyses. Regardless, the significant dissociation of NRXN3 proteoform and transcript expression uncovered here highlights the critical importance of proteoform-centric investigation in studies of neuronal wiring. Extensive single-cell sequencing studies uncovered transcriptomic profiles that predict functional properties of neuronal cells and the integration of transcriptomic and electrophysiological properties can identify neuronal subtypes (Fuzik et al., 2016; Tasic et al., 2018). However, attempts to correlate anatomical, electrophysiological, and transcriptome information across subclasses of PV-interneurons in the mouse hippocampus concluded that single-cell transcriptomes only weakly predict morphologically defined interneuron identities (Que et al., 2021). This apparent disconnect of molecular and anatomical features may arise from developmental specification of cell morphologies (Lim et al., 2018) and/or the extensive post-transcriptional regulation at the level of alternative splicing, and mRNA translation (Furlanis et al., 2019; Mauger et al., 2016; Mayr, 2017; Wang et al., 2019). The workflow established here demonstrates the feasibility and value of combining transcriptomic approaches with targeted proteome level analyses and might help design future studies interrogating molecular mechanisms of synapse development.

## STAR★Methods

### Key resources table

**Table:** 

REAGENT or RESOURCE	SOURCE	IDENTIFIER
**Antibodies**		

Rabbit polyclonal anti-beta Actin	Abcam	Cat# ab8227; RRID: AB_2305186; LOT# GR3314266-1
Mouse monoclonal anti-Calbindin	Swant	Cat# 300; RRID: AB_10000347; LOT# 17 (F)
Goat polyclonal anti-Calretinin	Swant	Cat# CG1; RRID: AB_10000342; LOT# 1§.1
Mouse monoclonal anti-CamKIIα (6G9)	Thermo Fisher Scientific	Cat# Ma1-048; RRID: AB_325403; LOT# TH269517
Goat polyclonal anti-Cannabinoid Receptor 1	Nittobo Medical	Cat# MSFR100600; RRID: AB_2571592
Mouse monoclonal anti-Cannabinoid Receptor 1 (IMG-3C2)	Immunogenes	Cat# IMG-CB1R-mAb001; LOT# CJ03
Guinea pig polyclonal anti-Cholecystokinin	Synaptic Systems	Cat# 438004; LOT# 1-1
Guinea pig polyclonal anti-GABA(A) Receptor α 1	Nittobo Medical	Cat# MSFR101540; RRID: AB_2571572
Mouse monoclonal anti-GAD67 (1G10.2)	Millipore	Cat# MAB5406; RRID: AB_2278725; LOT# 3015328
Rabbit polyclonal anti-GAPDH	Enogene	Cat# E1C604; LOT# R14Q12
Mouse monoclonal anti-Gephyrin (mAb7a)	Synaptic Systems	Cat# 147011; RRID: AB_887717; LOT# 147011/54
Mouse monoclonal anti-Gephyrin (mAb7a)	Synaptic Systems	Cat# 147021; RRID: AB_2232546; LOT# 147021/15
Rabbit monoclonal anti-HA (C29F4)	Cell Signaling	Cat# 3724; RRID: AB_1549585; LOT# 9
Rat monoclonal anti-HA (3F10)	Roche	Cat# 11867431001; RRID: AB_390919; LOT# 34502100
Guinea pig polyclonal anti-MAP2	Synaptic Systems	Cat# 188004; RRID: AB_2138181; LOT# 2-26
Mouse monoclonal anti-MAP2 (198A5)	Synaptic Systems	Cat# 188011; RRID: AB_2147096; LOT# 1-10
Chicken polyclonal anti-Neurexin	Nguyen et al. (2016)	N/A
Rabbit polyclonal anti-Neurexin	Muhammad et al. (2015)	N/A
Rabbit polyclonal anti-Neuroligin	Taniguchi et al. (2007)	N/A
Rabbit monoclonal anti-nNOS (C7D7)	Cell Signaling	Cat# 4231; RRID: AB_2152485; LOT# 2
Goat polyclonal anti-Parvalbumin	Swant	Cat# PVG214; RRID: AB_10000345
Mouse monoclonal anti-PSD-95 (7E3)	Santa Cruz	Cat# sc32290; RRID: AB_628114; LOT# J1509
Guinea pig polyclonal anti-PSD-95	Nittobo Medical	Cat# MSFR105180; RRID: AB_2571612
Goat polyclonal anti-Somatostatin (D20)	Santa Cruz	Cat# sc7819; RRID: AB_2302603; LOT# L1611
Guinea pig polyclonal anti-proSomatostatin	This study	GenBank: #BC010770.1
Mouse monoclonal anti-Synaptotagmin 2	Zebrafish International Resource Center	Cat# znp-1; RRID: AB_10013783
Mouse monoclonal anti-V5 (SV5-PK1)	Biorad	Cat# MCA1360; RRID: AB_322378; LOT# 148239
Guinea pig polyclonal anti-vGAT	Synaptic Systems	Cat# 131004; RRID: AB_887873; LOT# 2-42
Goat polyclonal anti-vGAT	Nittobo Medical	Cat# MSFR106130; RRID: AB_2571623
Guinea pig polyclonal anti-vGlut1	Millipore	Cat# AB5905; RRID: AB_2301751; LOT# 3308226
Rabbit polyclonal anti-VIP	Immunostar	Cat# 20077; RRID: AB_572270; LOT# 1513001

**Bacterial and virus strains**		

DH5α competent cells	Thermo Fisher Scientific	Cat# 18265017

**Chemicals, peptides, and recombinant proteins**		

Actinomycin D	Sigma Aldrich	Cat# A1410; CAS 50-76-0
Cycloheximide	Sigma Aldrich	Cat# C1988; CAS 66-81-9
Picrotoxin	Sigma Aldrich	Cat# P1675
D-AP5	Tocris Bioscience	Cat# 0106
NBQX	Tocris Bioscience	Cat# 1044
Tetrodotoxin (TTX)	Alomone Labs	Cat# T-550
^3^H-Ethanolamine Hydrochloride	Hartmann Analytic	Cat# ART0216

**Critical commercial assays**		

Dual-Luciferase Reporter Assay	Promega	Cat# E1910
RNAscope Fluorescent Multiplex Assay (individual probes listed in Method Details)	Advanced Cell Diagnostics	Cat# 320850

**Deposited data**		

Targeted Proteomic Data	This study	PanoramaWeb: https://panoramaweb.org/__r8753/project-begin.view
Shotgun Mass-Spectronomy Data	This study	PRIDE database: Project https://doi.org/10.6019/PXD031379

**Experimental models: Cell lines**		

Neuro2A Neuroblastoma cells	ATCC	#CCL-131
HEK293T human embryonic kidney cells	Takara	#632273
COS-7 monkey kidney cells	ATCC	#CRL-1651

**Experimental models: Organisms/strains**		

Mouse: Nrxn3-AS5KO (B6-Nrxn3<em2Schei>)	This study	N/A
Mouse: Nrxn3-HA (B6-Nrxn3<tm2(HA)Schei>)	This study	N/A
Mouse: LoxP-ChR2 (B6.Cg-Gt(ROSA)26Sortm32(CAG-COP4^∗^H134R/EYFP)Hze/J)	The Jackson Laboratory; Madisen et al. (2012)	RRID: IMSR_JAX:024109
Mouse: PV-Cre (B6.129P2-Pvalb<tm1(cre)Arbr>/J)	The Jackson Laboratory; Hippenmeyer et al. (2005)	RRID: IMSR_JAX:017320
Mouse: Sst-Cre (Sst<tm2.1(cre)Zjh>/J)	The Jackson Laboratory; Taniguchi et al. (2011)	RRID: IMSR_JAX:013044
Mouse: RjOrl:Swiss (CD-1)	Janvier Labs	https://www.janvier-labs.com/en/fiche_produit/swiss_mouse/

**Oligonucleotides**		

For primer sequences, please see Table S4	This study	N/A

**Recombinant DNA**		

Plasmid: psiCHECK-2	Promega	Cat# C8021
Plasmid: pAPtag-5	GenHunter	Cat# QV5
Plasmid: pDisplay-AP-CFP-TM	Alice Ting Lab	RRID: Addgene_20861
Plasmid: pET29b(+)	Novagen	Cat# 69872
Plasmid: pDisplay-V5-Nxph1	This study	submitted to Addgene
Plasmid: FAM19A1-V5	This study	submitted to Addgene
Plasmid: FAM19A2-V5	This study	submitted to Addgene
Plasmid: pDisplay-EGFP-2A-HA-Nrxn3-alpha- 2(-)/3(+)/4(+)/6(-)-GPI	This study	submitted to Addgene
Plasmid: pDisplay-EGFP-2A-HA-Nrxn3-alpha- 2(-)/3(+)/4(+)/6(+)	This study	submitted to Addgene
Plasmid: Nrxn3α-AS4- (pDisplay-EGFP-2A-HA-Nrxn3-alpha-2(+)/3(+)/4(-)/6(+))	Nguyen et al. (2016)	submitted to Addgene
Plasmid: Nrxn3α-AS4+ (pDisplay-EGFP-2A-HA-Nrxn3-alpha-2(+)/3(+)/4(+)/6(+))	Nguyen et al. (2016)	submitted to Addgene

**Software and algorithms**		

Metamorph v7.8.11.0	Molecular Devices	https://www.moleculardevices.com/products/cellular-imaging-systems/acquisition-and-analysis-software/metamorph-microscopy; RRID:SCR_002368
Igor Pro v6.31	WaveMetrics	https://www.wavemetrics.com/products/igorpro; RRID:SCR_000325
SparkControl v2.3	Tecan	https://lifesciences.tecan.com/multimode-plate-reader?p=tab--3
Xcalibur v4.4.16.14	Thermo Scientific	https://www.thermofisher.com/order/catalog/product/OPTON-30965
StepOne v2.3	Applied Biosystems	https://www.thermofisher.com/ch/en/home/technical-resources/software-downloads/StepOne-and-StepOnePlus-Real-Time-PCR-System.html; RRID:SCR_014281
ImageJ v2.1.0/1.53c	NHI	https://imagej.nih.gov/ij/download.html; RRID:SCR_003070
Synapse Counter PlugIn ImageJ	Faissner Lab	https://github.com/SynPuCo/SynapseCounter; PMID: 27615741
Progenesis QI v2.0	Nonlinear Dynamics	https://www.nonlinear.com/progenesis/qi-for-proteomics/; RRID: SCR_018923
MASCOT, v2.4.1	Matrix Science	https://www.matrixscience.com; RRID: SCR_014322
Prism v7 / v9	GraphPad	https://www.graphpad.com/scientific-software/prism/; RRID: SCR_002798
Skyline v21.1.0.146	MacCoss Lab	https://skyline.gs.wawashingt.edu/labkey/project/home/softwars/Skyline/begin.view; RRID: SCR_014080
Safe Quant v2.3.2	Schmidt Lab	https://github.com/eahrne/SafeQuant
Max Quant v.1.6.2.3	Cox Lab	https://www.maxquant.org
SpectroDive v10	Biognosys	https://biognosys.com/software/spectrodive/
Adobe Illustrator CC	Adobe	http://www.adobe.com/products/illustrator.html; RRID: SCR_010279
Adobe Photoshop CC	Adobe	https://www.adobe.com/products/photoshop.html; RRID: SCR_014199
CRISPOR software	Haeussler Lab	http://crispor.tefor.net; PMID: 29762716
Stimfit 0.15.8	Schmidt-Hieber Lab	https://github.com/neurodroid/stimfit; RRID: SCR_016050
ZEN software	Zeiss Microscope	https://www.zeiss.com/microscopy/int/products/microscope-software/zen.html; RRID: SCR_013672
Omero	Open Microscopy Environment	http://www.openmicroscopy.org/site/products/omero; RRID: SCR_002629

### Resource availability

#### Lead contact

Further information and requests for resources and reagents should be directed to and will be fulfilled by the lead contact, Peter Scheiffele (peter.scheiffele@unibas.ch).

#### Materials availability

This study has generated antibodies, plasmids and mouse lines, which are listed in the key resource table. Plasmids have been deposited to Addgene, antibodies and mouse lines will be made available upon request.

### Experimental model and subject details

#### Animals

All procedures involving animals were approved by and performed in accordance with the guidelines of the Kantonales Veterinäramt Basel-Stadt, Switzerland. Mice were maintained on 12-hour light/dark cycle with water and food available *ad libitum*. Male and female mice were used, unless indicated otherwise. Age of the animals is indicated in individual experiments, in general mice were used at P25-30 for biochemical and immunohistochemical analysis and at 6-8 weeks for electrophysiological recordings.

The following mice strains were used in this study: LoxP-ChR2 (JAX: 024109; Madisen et al., 2012), PV-Cre (JAX: 017320; Hippenmeyer et al., 2005) and SST-Cre (JAX: 013044; Taniguchi et al., 2011) mice were obtained from Jackson Laboratories, RjOrl:Swiss mice (CD-1) were from Janvier Labs, for generation of *Nrxn3*^*ΔEx24*^ knock-out and *Nrxn3 AS5*^*HA*^ knock-in mice see STAR Methods details. All mouse lines were maintained on a C57BL6/J strain background.

In general, het/het breeding schemes were used for *Nrxn3*^*ΔEx24*^ knock-out and *Nrxn3 AS5*^*HA*^ knock-in mice. For Cre mediated ChR2 expression, homozygous PV- or SST-Cre animals (heterozygous for *Nrxn3*^*ΔEx24*^) were mated with homozygous LoxP-ChR2 animals (heterozygous for *Nrxn3*^*ΔEx24*^), and experiments were performed with 6-8 week old mice heterozygous for PV- and SST-Cre or LoxP-ChR2.

#### Cell lines

Neuro2a (ATCC, #CCL-131), HEK293T (Takara, #632273) and Cos7 (ATCC, #CRL-1651) cells were maintained in DMEM (Sigma D5796) containing glucose (4500mg/l) supplemented with 10% fetal calf serum (FCS) and penicillin/streptomycin (Sigma P4333) at 37°C/5% CO_2_.

#### Primary cells

Cortical (from CD-1 mice) and hippocampal (from C57Bl6J *Nrxn3 AS5*^*HA*^ mice) neuronal cells were maintained in neurobasal medium (Gibco 21103) containing 2% B27 supplement (Gibco 17504-044), 1% Glutamax (Gibco 35050-038), and 1% penicillin/streptomycin at 37°C / 5% CO_2_. Isolation of primary cells is described in the Method Details.

### Method details

#### Generation of transgenic mouse models

The *Nrxn3 AS5*^*HA*^ knock-in allele was obtained by CRISPR/Cas9-mediated gene editing in electroporated mouse embryos. The target sequence atgtccatgtaagggcggca(cgg) (PAM sequence in brackets) was selected with CRISPOR software (Concordet and Haeussler, 2018). Sequences were inserted into the Cas9-generated double-stranded DNA break by homologous recombination using an asymmetric single-stranded donor DNA (Richardson et al., 2016) *cttccttacagccagaagctcta*ttgcagct**tacccatacgatgttcctgactatgcg**ggc**tatccctatgacgtcccggactatgca**ggaacagccagaagctctaacgcggcgagatcacta*cgtgccgcccttacatggacatggcgactcacttacacact* (dual HA epitope tag sequence in bold, 5’ and 3’ homology arms in italics).

*Nrxn3*^*ΔEx24*^ knock-out mice were generated by CRISPR/Cas9-mediated editing in microinjected embryos. Two gRNAs targeting sequences flanking exon 24 were selected: intron 23 (INT23) gcagtagtacaaatcatggg(tgg) and intron 24 (INT24) gagagcaaataataccaata(agg) (PAM sequences in brackets).

Embryos were obtained from C57BL/6J female mice. Mice underwent ovulation induction by i.p. injection of 5 IU equine chorionic gonadotrophin (PMSG; Folligon–InterVet), followed by i.p. injection of 5 IU human chorionic gonadotropin (Pregnyl–Essex Chemie) 48 h later. For the recovery of zygotes, C57BL/6J females were mated with males of the same strain immediately after the administration of human chorionic gonadotropin. All zygotes were collected from oviducts 24 h after the human chorionic gonadotropin injection and were then freed from any remaining cumulus cells by a 1–2 min treatment of 0.1% hyaluronidase (Sigma-Aldrich) dissolved in M2 medium (Sigma-Aldrich).

For electroporation, the zona pellucida was partially removed by brief treatment with acid Tyrode’s solution and the embryos were washed and briefly cultured in M16 (Sigma) medium at 37°C and 5% CO_2_. Electroporation with a mixture of ssDNA oligonucleotide targeting template, 16μM cr:trcrRNA hybrid targeting *Nrxn3* and 16μM Cas9 protein (all reagents from IDT) was carried out using 1mm gap electroporation cuvette and the ECM830 electroporator (BTX Harvard Apparatus). Two square 3 ms pulses of 30V with 100 ms interval were applied as previously described (Chen et al., 2016).

For microinjection, mouse embryos were cultured in M16 (Sigma-Aldrich) medium at 37°C and 5% CO_2_. For manipulation, embryos were transferred into M2 medium. Microinjections were performed using a microinjection system comprised of an inverted microscope equipped with Nomarski optics (Nikon), a set of micromanipulators (Narashige), and a FemtoJet microinjection unit (Eppendorf). Injection solution containing: Cas9 protein (IDT) 100ng/μl (60μM), cr:trcrRNA INT23 (IDT) 50μM, cr:trcrRNA INT24 (IDT) 50μM, LoxP_INT_23 oligo 10ng/ul, LoxP_INT_24 oligo 10ng/ul was microinjected into the male pronuclei of fertilized mouse oocytes until 20-30% distension of the organelle was observed.

Embryos that survived the manipulations were transferred on the same day into the oviducts of 8–16-wk-old pseudopregnant Crl:CD1 (ICR) females (0.5 d used after coitus) that had been mated with sterile genetically vasectomized males the day before embryo transfer (Haueter et al., 2010). Pregnant females were allowed to deliver and raise their pups until weaning age.

Selected founder animals were bred to C57BL/6J partners and then further back-crossed to C57BL/6J mice for >8 generations.

#### Cell culture

HEK293T, Cos7 and Neuro2A cells were maintained in DMEM (Sigma D5796) containing glucose (4500mg/l) supplemented with 10% fetal calf serum (FCS) and penicillin/streptomycin (Sigma P4333) at 37°C / 5% CO_2_. Transfections were performed with Gibco™ Opti-MEM™ reduced serum medium and FuGENE® 6 transfection reagent according to the manufacturer instructions. HEK293T cells for surface stainings were grown on gelatine coated (0.1% in H_2_O) coverslips.

Cortical cultures were prepared from E16.5 mouse embryos. Neocortices were dissociated by addition of papain (130 units, Worthington Biochemical LK003176) for 30 min at 37°C. Cells were maintained in neurobasal medium (Gibco 21103) containing 2% B27 supplement (Gibco 17504-044), 2mM Glutamax (Gibco 35050-038), and 1% penicillin/streptomycin at 37°C / 5% CO_2_. Cortical cultures were treated for 4 hours at day in vitro 12 with transcription and/or translation inhibitors (10μg/ml actinomycin D, Sigma, A1410; 25μg/ml cycloheximide, Sigma, C1988; stocks dissolved at 1000x in DMSO).

For hippocampal cultures, Hippocampi from P0 mice were dissected, trypsinized for 10 min in 0.05% trypsin (Gibco 25300) buffered with 10mM HEPES (Gibco 15630) at 37°C, washed 3x with HBSS (Gibco 14025) containing additional 10mM HEPES and triturated using a fire-polished glass Pasteur pipette. Cells were plated at a density of 10,000-12,000 cells per cm^2^ on poly-D-lysine (Sigma P7886) coated glass coverslips in DMEM containing 1% penicillin/streptomycin and 10% fetal bovine serum. 4‑6 h after plating, medium was changed to serum-free Neurobasal supplemented with 2 mM GlutaMax, 1% B27 supplement and 1% penicillin/streptomycin. Cells were then maintained at 37°C / 5% CO_2_.

#### Biochemical procedures

Cell lysates were obtained from dissected brain regions of P2-P60 homozygous and heterozygous *Nrxn3 AS5*^*HA*^ knock-in mice or *wild-type* littermates by homogenization in 50mM Tris-HCl pH7.5, 150mM NaCl, 1% Triton-X100, 1mM EDTA and protease inhibitors (Roche cOmplete™ mini). Lysates were centrifuged for 10 min, 16,000g at 4°C and supernatants analyzed by Western-Blotting.

For membrane fractionation mouse brain tissue (P25-P30) was homogenized in 0.32M sucrose, 50mM HEPES pH7.4 supplemented with protease inhibitors (Roche complete™ mini) using a glass-teflon homogenizer. Extracts were centrifuged for 5 min at 16,000g. Subsequently, supernatants (= “input fractions”) were centrifuged for 60 min at 100,000g (TLA55 rotor, Optima™ MAX-XP Ultracentrifuge) and pelleted membranes were re-suspended in high salt buffer (1M NaCl, 10mM EDTA, protease inhibitors, pH 7.4). Membranes were centrifuged for 60 min at 100,000g and salt-washed membranes were re-suspended in 150mM NaCl, 10mM EDTA, pH 7.0. Proteins were precipitated by methanol/chloroform method and analyzed by Western Blotting. Membrane fractionation from HEK293 cells (transiently transfected with AP-eGPI or AP-NRXN3Ex24 expression vectors, generated by standard molecular cloning methods inserting endogenous GPI propeptide encoding sequence (eGPI) or coding sequence of *Nrxn3* Exon 24 into pAPtag-5) followed the same protocol, except that cell extracts were homogenized by passing through a 28G needle and using 2M KCl in 10mM HEPES, pH 7.4, 10mM EDTA, protease inhibitors for salt wash. For radioactive ethanolamine labeling, transfected HEK293 cells were incubated 4 hours after transfection with ^3^H-Ethanolamine (100μCi) overnight at 37°C/5% CO_2_. Cells were lysed in 1ml IP-Buffer (50mM Tris-HCl pH 7.5, 150mM NaCl, 10% Glycerol, 1% Triton-X100, 0.1% SDS, protease inhibitors) and cell lysate were centrifuged for 15 min at 16’000g, 4°C. The supernatant was transferred to a new tube and incubated overnight at 4°C with 20μl anti-HA coupled magnetic beads (Pierce, 88837). Beads were washed 4x in IP-Buffer, denatured and analyzed by SDS gel electrophoresis followed by fixation (30 mins, 25% Isopropanol, 10% Acetic Acid), 30 min incubation with Amplify Fluorographic reagent (GE Healthcare Life Sciences), and exposed to X-ray film (Amersham Hyperfilm MP, GE Healthcare Life Sciences) after drying.

#### Immunohistochemistry procedures

Mice (postnatal day 25 to 30) were deeply anesthetized with ketamine/xylazine (100/10mg/kg i.p.) and trans-cardially perfused with fixative (4% paraformaldehyde and 15% picric acid in 100mM phosphate buffer, pH 7.4). Alternatively, to optimize detection of synaptic antigens, 9% glyoxal (Richter et al., 2018) in 8% acetic acid (pH 4) was used as fixative. After perfusion, brains were post-fixed overnight in fixative at 4°C, washed 3 times with PBS, and kept overnight at 4°C in 30% Sucrose in 100mM phosphate buffer before cryo-protection in OCT. Coronal brain slices were cut at 30μm (50μm for glyoxal fixed brains) with a Cryostat (Microm HM560, Thermo Scientific). For immunohistochemistry, brain sections were incubated for 0.5-1hr in blocking solution containing 0.1% Triton X-100 and 10% normal donkey serum in PBS. Slices were incubated with primary antibodies in blocking solution at 4°C two times overnight and washed three times in PBS containing 0.05% Triton X-100, followed by incubation for 1-2 hours at room temperature with secondary antibodies. Sections were washed three times in PBS containing 0.05% Triton X-100 and one time with PBS before mounting onto microscope slides with Fluoromount-G (SouthernBiotech, 0100-01). Hoechst dye was co-applied during washing at a final concentration of 0.5 μg/ml.

Hippocampal cells in dissociated culture or transfected Cos7 (transiently transfected with AP-eGPI or AP-NRXN3Ex24 expression vectors) and HEK293T cells (transiently transfected with pDisplay constructs expressing membrane bound CFP (pDisplay-AP-CFP-TM), or NRXN3AS5+ (pDisplay-EGFP-2A-HA-Nrxn3-alpha-2(-)/3(+)/4(+)/6(+)-GPI) and NRXN3AS5- (pDisplay-EGFP-2A-HA-Nrxn3-alpha-2(-)/3(+)/4(+)/6(+))) were fixed for 10 min using 4% PFA / 4% sucrose in 0.1M phosphate buffer pH 7.4 at room temperature (RT) and washed 3x with PBS. Hippocampal cells were quenched 10 min with 0.1M glycine and blocked for 1h at RT with 10% normal donkey serum and 0.1% Triton-X100 in PBS. Primary antibodies were applied overnight at 4°C in blocking solution. After 4 washes with PBS, fluorophore-coupled secondary antibodies were applied 60 min at RT. Cells were washed three times with PBS before mounting as described above for brain sections. For surface labeling of Cos7 and HEK293T cells, cells were blocked for 1h at RT with 5% milk powder in PBS and primary antibodies were applied in 1% BSA / PBS overnight at 4°C, subsequent steps were as described above for hippocampal cells.

The following antibodies were used in this study: rabbit polyclonal anti-β-actin (Abcam; Cat# ab8227; RRID: AB_2305186; LOT# GR3314266-1), mouse monoclonal anti-calbindin (Swant; Cat# 300; RRID: AB_10000347; LOT# 17 (F)), goat polyclonal anti-calretinin (Swant; Cat# CG1; RRID: AB_10000342; LOT# 1§.1), mouse monoclonal anti-CamKII alpha (Thermo Fisher Scientific; 6G9; Cat# Ma1-048; RRID: AB_325403; LOT# TH269517), mouse monoclonal anti-cannabinoid receptor 1 (Immunogene; IMG-3C2; Cat# IMG-CB1R-mAb001; LOT# CJ03), guinea pig polyclonal anti-cholecystokinin (Synaptic Systems, Cat# 438004; RRID: AB_2814938; LOT# 1-1), mouse monoclonal anti-GAD67 (Millipore; 1G10.2; Cat# MAB5406; RRID: AB_2278725; LOT# 3015328), rabbit polyclonal anti-GAPDH (Enogene; Cat# E1C604; LOT# R14Q12), mouse monoclonal anti-gephyrin (Synaptic Systems; mAb7a; Cat# 147021; RRID: AB_2232546; LOT# 147021/15), mouse monoclonal anti-gephyrin (Synaptic Systems; mAb7a; Cat# 147011; RRID: AB_887717; LOT# 147011/54), rat monoclonal anti-HA (Roche; 3F10; Cat# 11867431001; RRID: AB_390919; LOT# 34502100), rabbit monoclonal anti-HA (Cell Signaling; 3724; Cat# 3724; RRID: AB_1549585; LOT# 9), guinea pig polyclonal anti-MAP2 (Synaptic Systems; Cat# 188004; RRID: AB_2138181; LOT# 2-26), mouse monoclonal anti-MAP2 (Synaptic Systems; 198A5; Cat# 188011; RRID: AB_2147096; LOT# 1-10), rabbit polyclonal anti-neurexin (Muhammad et al., 2015), chicken anti-neurexin (Nguyen et al., 2016), rabbit anti-neuroligin (Taniguchi et al., 2007), rabbit monoclonal anti-nNOS (Cell Signaling; C7D7; Cat# 4231; RRID: AB_2152485; LOT# 2), goat polyclonal anti-parvalbumin (Swant, Cat# PVG214; RRID: AB_10000345), mouse monoclonal anti-PSD95 (Santa Cruz; 7E3; Cat# sc32290; RRID: AB_628114; LOT# J1509), goat polyclonal anti-somatostatin (Santa Cruz; Cat# sc7819; RRID: AB_2302603; LOT# L1611), mouse monoclonal anti-Synaptotagmin 2 (Zebrafish International Resource Center; Cat# znp-1; RRID: AB_10013783), mouse monoclonal anti-V5 (Biorad; SV5-PK1; Cat# MCA1360; RRID: AB_322378; LOT# 148239), guinea pig polyclonal anti-vGAT (Synaptic Systems; Cat# 131004; RRID: AB_887873; LOT# 2-42), guinea pig polyclonal anti-vGlut1 (Millipore; Cat# AB5905; RRID: AB_2301751; LOT# 3308226), rabbit polyclonal anti-VIP (Immunostar; Cat# 20077; RRID: AB_572270; LOT# 1513001), guinea pig anti-somatostatin (raised in the present study against amino acid residues 35-88 of mouse pro-somatostatin, GenBank: #BC010770.1), goat anti-cannabinoid receptor 1 (Nittobo Medical, MSFR100600; RRID: AB_2571592), guinea pig anti-GABAA receptor alpha 1 (Nittobo Medical, MSFR101540; RRID: AB_2571572), goat anti-vGAT (Nittobo Medical, MSFR106130; RRID: AB_2571623), guinea pig anti-PSD95 (Nittobo Medical, MSFR105180; RRID: AB_2571612).

Fluorophore-conjugated secondary antibodies were from Life Technologies (Alexa Fluor 568 goat anti-rat #A11077, Alexa Fluor 488 donkey anti-mouse #A21202, Alexa Fluor 647 donkey anti-goat #A21447) and Jackson ImmunoResearch (Cy2 donkey anti-goat #705-225-147, Cy2 donkey anti-guinea pig #706-225-148, Cy3 donkey anti-rabbit #711-165-152, Cy3 donkey anti-mouse #715-165-151, Cy3 donkey anti-goat #705-165-147, Alexa Fluor 488 donkey anti-guinea pig #706-545-148, Cy5 donkey anti-mouse #715-175-511, Cy5 donkey anti-rabbit #711-175-152, Alexa Fluor 647 donkey anti-guinea pig #706-605-148). Hoechst 33342 dye (Sigma #B2261) was used for nuclear staining. Secondary antibodies coupled to horse radish peroxidase (HRP) were from Jackson ImmunoResearch (goat anti-rabbit HRP #111-035-003; goat anti-rat HRP #112-035-143). For enhanced chemiluminescence detection, WesternBright ECL kit (Advansta #K12045-D20) and WesternBright Quantum (Advansta #K-12042-D20) were used. Signals were acquired using an image analyzer (Bio-Rad, ChemiDoc MP Imaging System and Li-Cor, Odyssey) and images were analyzed using ImageJ.

Images from brain sections and cultured cells were acquired on a confocal microscope (Zeiss LSM700), using 20x, 40x and 63x Plan-Apochromat objectives (numerical aperture 0.45, 1.30 and 1.40, respectively) and were then processed in Fiji and Omero. Quantitative analysis of cellular markers was performed manually, quantification of synapses in hippocampal culture and for synaptic markers in brain slices was performed with synapse counter plug-in (Dzyubenko et al., 2016). Analysis parameters were optimized according to the synapse counter plug-in guidelines, defining minimum and maximum puncta size and using Otsu thresholding.

#### Immunoelectron microscopy

All immunohistochemical incubations were performed at room temperature. For silver-enhanced pre-embedding immunogold electron microscopy, sections were dipped in 10% normal goat serum/PBS for 30 mins, incubated overnight with rabbit anti-HA antibody (Cell Signaling, 1:1000) diluted with 0.1% TritonX-100/PBS, and subjected to silver-enhanced immunogold labeling using anti-rabbit IgG conjugated with 1.4 nm gold particles (Nanogold; Nanoprobes, USA) and R-Gent SE-EM Silver Enhancement Reagents (Aurion, Netherlands). Sections were further treated with 1% osmium tetroxide and 2% uranyl acetate, and embedded in Epon812. Ultrathin sections (100 nm in thickness) were prepared with an ultramicrotome (Leica, Wien, Austria), and photographs were taken with an H7100 electron microscope (Hitachi, Tokyo, Japan). The density and distribution of immunogold particles were analyzed on electron micrographs using MetaMorph software (Molecular Devices). The density of HA on symmetrical and asymmetrical synapses was calculated by measuring the number of immunogold particles. Perpendicular distribution of HA was examined by sampling synaptic profiles whose presynaptic and postsynaptic membranes were cut perpendicularly to the plane of the synaptic cleft, and by measuring the distance from the midline of the synaptic cleft to the center of immunogold particles.

#### RNA analysis

For RNA isolation, brain tissue or cultured cells were dissected or washed in ice-cold PBS respectively, homogenized in 1 ml TRI Reagent (Sigma T9424) and thoroughly mixed with 200 μl chloroform (Sigma 2432). Samples were centrifuged at 16’000 g, 4°C for 15 min. The aqueous phase was used for RNA purification with the RNeasy Plus Mini kit (Qiagen 74134) or RNeasy Micro kit (Qiagen 74034) following the manufacturer’s instructions, including on-column DNase-treatment to remove traces of genomic DNA. 0.5μg of total RNA was reverse transcribed using random hexamers (Promega C1181) or Oligo(dT)_15_ primer (Promega C1101) for flanking primer analysis and ImProm II reverse transcriptase (Promega A3802).

For qPCR assays, two technical replicates were run per experiment and the mean was calculated. The mRNA levels were normalized to *gapdh* mRNA. qPCR assays were analyzed with StepOne software. Flanking primer PCRs were run with FirePol Master mix (Solis BioDyne, 04-11-00125) on mRNA reverse transcribed with Oligo(dT)_15_ primers, PCR cycle numbers were carefully titrated to ensure correct amplification range and avoid signal saturation. DNA oligonucleotides used with SYBR Green-based real-time PCR and for flanking primer PCRs are listed in Table S4.

Sashimi plots were generated from published RNA-Seq data (Furlanis et al., 2019) using the MISO software package (Katz et al., 2010).

For detection of Nrxn3 binding partner mRNAs, snap frozen brains were cut on a cryostat into 13μm sections, adhered to Superfrost ultra plus slides (Thermo Scientific) and stored at −80 °C. Sections were fixed for 30 min in 4% PFA before being processed using the RNAscope Fluorescent Multiplex Kit (ACD) according to the manufacturer’s instruction. The following probes were used: *Nrxn3* (C1, Ref# 525951, Lot# 21260B), *Nxph1* (C2, Ref# 463401-C2, Lot# 21270A) and *Fam19a2* (C2, Ref# 452631-C2, Lot# 21312A). Probes were combined as *Nrxn3/Nxph1* or *Nrxn3/Fam19a2*. Amp-4-Alt C was used for all combinations. Sections were imaged with a confocal microscope (Zeiss LSM700), using 20x and 63x Plan-Apochromat objectives (numerical aperture 0.45 and 1.40, respectively) and were then processed in Fiji and Omero.

For in situ hybridization against somatostatin mRNA, complementary DNA fragments encoding mouse somatostatin (133-408 bp; NM_012659) were subcloned into the Bluescript II plasmid vector. Digoxigenin (DIG)-labeled cRNA probes were prepared as previously described (Yamasaki et al., 2001).

Sections were acetylated with 0.25% acetic anhydride in 0.1M triethanolamine-HCl (pH 8.0) for 10 min and prehybridization was performed for 1 h in hybridization buffer (50% formamide, 50mM Tris-HCl (pH 7.5), 0.02% Ficoll, 0.02% polyvinylpyrrolidone, 0.02% bovine serum albumin, 0.6M NaCl, 200 μg/mL of tRNA, 1mM EDTA and 10% dextran sulfate). Hybridization was performed at 63.5 °C for 12 h in hybridization buffer supplemented with cRNA probes at a dilution of 1:1000. Post-hybridization washing was done at 61 °C successively with 5x standard sodium citrate (SSC) for 30 min, 4x SSC containing 50% formamide for 40 min, 2x SSC containing 50% formamide for 40 min, and 0.1x SSC for 30 min. Sections were incubated at room temperature in NTE buffer (0.5M NaCl, 0.01M Tris-HCl (pH 7.5) and 5mM EDTA) for 20 min, 20mM iodoacetamide in NTE buffer for 20 min, and TNT buffer (0.1M Tris-HCl (pH 7.5) and 0.15M NaCl) for 20 min.

For immunohistochemical detection of DIG, sections were blocked with DIG blocking solution (TNT buffer containing 1% blocking reagent (Roche Diagnostics, Basel, Switzerland) and 4% normal sheep serum) for 30 min and 0.5% TSA blocking reagent (PerkinElmer, Waltham, MA, USA) in TNT buffer for 30 min. Then, sections were incubated with peroxidase-conjugated anti-DIG (1:1000, 2 h; Roche Diagnostics) for fluorogenic detection. After the TNT wash twice for 15 min each, fluorogenic detection for single fluorescent *in situ* hybridization was performed using the Cy3-TSA plus amplification kit (PerkinElmer) followed by immunofluorescence detection for somatostatin protein.

#### Luciferase assays

*Nrxn3* 3’UTR sequences were amplified from cDNA and inserted into the dual luciferase psiCHECK-2 vector (Promega) after the Renilla luciferase open reading frame. The resulting 3’UTR sequences are listed in Table S4. Neuro2a cells were plated at 20,000 cells/well in a 96 well plate. After 24h, cells were transfected with 50 ng dual luciferase construct containing different 3’UTRs of *Nrxn3*. psiCHECK-2 (containing only Firefly luciferase and Renilla luciferase) was used as a negative control. Cells were collected after 24 hours for processing using the Dual-Luciferase reporter assay kit (Promega, E1910) or for purification of RNA. Renilla and firefly luciferase activity was measured using a Tecan Sparks plate reader and Renilla luciferase activity was normalized to firefly activity. mRNA levels were quantified by RT-qPCR using primers against Renilla and Firefly cDNA. Absence of DNA contamination was controlled by processing negative control samples where reverse transcriptase was omitted from the protocol.

#### Neurexin complex purifications

Hippocampi from P28 animals (*wild-type* or *Nrxn3AS5*^*HA/HA*^) were dissected, snap frozen and stored at -80°C until use. For immunoprecipitation, hippocampi were homogenized by 30 strokes in glass-homogenizer in 2 mL IP-buffer (50 mM Tris–HCl, pH7.5, 150 mM NaCl, 10% glycerol, protease inhibitors, 1% Triton X-100, and 2 mM CaCl_2_) for 2x hippocampi from one animal. Homogenates were centrifuged at 16’000 x g for 15 min at 4°C. Supernatants were transferred to new tubes. For precipitation of NRXN3 AS5^HA^ lysates were incubated for 2 h at 4°C with rotation and centrifuged at 16’000 x g for 5 min at 4°C. Supernatants were transferred to new tubes containing 18 μL of HA-magnetic beads (Pierce / Thermofisher). Samples were incubated for 6 h at 4°C with rotation. For precipitations with pan-NRX antibody 1 μg homemade affinity purified antibody or control rabbit IgG per 1 mL lysate were added. Samples were incubated for 4 h at 4°C with rotation and then centrifuged at 16’000 x g for 5 min at 4°C. Supernatants were transferred into new tubes and 18 μL of Protein-A-dynabeads (Thermofisher) were added to the samples. Samples were incubated for additional 2 h at 4°C with rotation. For both, HA- and pan-NRXN precipitations, beads were washed 3 x with IP-buffer, transferred to new tubes and then washed 3 x with IP-wash buffer (50 mM Tris–HCl, pH7.5, 150 mM NaCl, 10% glycerol, 0.1% Triton X-100, 2 mM CaCl_2_). Bound proteins were eluted from beads by incubation in 50 μL elution 1% SDS, 5 mM DTT for mass spectrometry or 40 μL Laemmli buffer (65°C for 10 min) for western blot analysis.

#### Mass spectrometry

##### Sample preparation

Eluted immuno-purified protein samples were mixed with one volume of 2x MS-lysis buffer (200mM Triethyl-ammonium bicarbonate (TEAB / Sigma), 10% SDS, 20 mM Tris-(2-carboxyethyl)-phosphin (TCEP / Sigma)) and incubated for 10 min at 95°C. Samples were cooled down to room temperature incubated for 30 mins with 20mM iodoacetamide (Sigma). Subsequently, samples were acidified by addition of phosphoric acid (1.2% final concentration), mixed 1:6 with S-trap buffer (90% methanol, 100 mM TEAB), loaded on S-trap micro columns (Protifi, NY, US), and washed 3 x with S-trap buffer. 20 μl of trypsin solution (2.5 μl trypsin (0.4 μg/μl Promega) in 50 mM TEAB)) was added on the columns and samples incubated 47°C for 1h. Peptides were eluted from the columns in 3 steps: 1x with 40 μL 50 mM TEAB, 1x with 40 μL 0.2% formic acid in H_2_O and 1x with 40 μL 0.2% formic acid in 50% acetonitrile. Eluted peptides were vacuum dried, re-suspended in 20 μL LC/MS-buffer (0.1% formic acid in water) and the peptide concentration determined using a SpectroStar nano Spectrophotometer (BMG Labtech, Germany). For targeted proteomics, to each peptide sample an aliquot of a heavy reference peptide mix containing chemically synthesized proteotypic peptides (Spike-Tides, JPT, Berlin, Germany) was spiked into each sample at a concentration of 2 fmol/ug of total peptide mass of heavy reference peptides per sample.

For targeted proteomics from brain tissue, dissected tissue was lysed and alkylated in lysis buffer (1% sodium deoxycholate (SDC), 0.1 M Tris-HCl, pH8.5, 15mM chloroacetamide, 10 mM TCEP) by homogenization with a 21G needle followed by ultra-sonication (10 cycles, Bioruptor, Diagnode). Samples were reduced for 10 min at 95 °C and digested by incubation with sequencing-grade modified trypsin (1/50, w/w; Promega, Madison, Wisconsin) overnight at 37°C. Peptides were cleaned up using iST cartridges (PreOmics, Munich) according to the manufacturer’s instructions. Samples were dried under vacuum and stored at -80 °C until further use. Samples were resuspended in 0.1% formic acid.

##### Shotgun LC-MS analysis of Neurexin immunoprecipitates

1 ug of peptides were subjected to LC–MS/MS analysis using a Q Exactive HF Mass Spectrometer fitted with an EASY-nLC 1000 (both Thermo Fisher Scientific) and a custom-made column heater set to 60°C. Peptides were resolved using a RP-HPLC column (75μm × 30cm) packed in-house with C18 resin (ReproSil-Pur C18–AQ, 1.9 μm resin; Dr. Maisch GmbH) at a flow rate of 0.2 μLmin-1. A linear gradient ranging from 5% buffer B to 45% buffer B over 60 minutes was used for peptide separation. Buffer A was 0.1% formic acid in water and buffer B was 80% acetonitrile, 0.1% formic acid in water. The mass spectrometer was operated in DDA mode with a total cycle time of approximately 1 s. Each MS1 scan was followed by high-collision-dissociation (HCD) of the 20 most abundant precursor ions with dynamic exclusion set to 30 seconds. For MS1, 3e6 ions were accumulated in the Orbitrap over a maximum time of 25 ms and scanned at a resolution of 120,000 FWHM (at 200 m/z). MS2 scans were acquired at a target setting of 1e5 ions, maximum accumulation time of 50 ms and a resolution of 15,000 FWHM (at 200 m/z). Singly charged ions, ions with charge state ≥ 6 and ions with unassigned charge state were excluded from triggering MS2 events. The normalized collision energy was set to 28%, the mass isolation window was set to 1.4 m/z and one microscan was acquired for each spectrum.

The acquired raw-files were imported into the Progenesis QI software (v2.0, Nonlinear Dynamics Limited), which was used to extract peptide precursor ion intensities across all samples applying the default parameters. The generated mgf-file was searched using MASCOT against a *mus musculus* database (SwissProt, www.uniprot.org, release date 26/09/2018) including all splice variants of interest plus commonly observed contaminants (in total 34,770 sequences) using the following search criteria: full tryptic specificity was required (cleavage after lysine or arginine residues, unless followed by proline); 3 missed cleavages were allowed; carbamidomethylation (C) was set as fixed modification; oxidation (M) and acetyl (Protein N-term) were applied as variable modifications; mass tolerance of 10 ppm (precursor) and 0.6 Da (fragments). The database search results were filtered using the ion score to set the false discovery rate (FDR) to 1% on the peptide and protein level, respectively, based on the number of reverse protein sequence hits in the dataset. Quantitative analysis results from label-free quantification were processed using the SafeQuant R package v.2.3.2. (Ahrné et al., 2016) (https://github.com/eahrne/SafeQuant/) to obtain peptide relative abundances. This analysis included data imputation using the knn algorithm, summation of peak areas per protein and LC-MS/MS run, followed by calculation of peptide abundance ratios. No global data normalization by equalizing the total peak/reporter areas across all LC-MS runs was performed for the AP-MS data to consider the low peptide amounts present in control samples. Only isoform specific peptide ion signals were considered for quantification. To meet additional assumptions (normality and homoscedasticity) underlying the use of linear regression models and t-Tests, MS-intensity signals were transformed from the linear to the log-scale. The summarized peptide expression values were used for statistical testing of between condition differentially abundant peptides. Here, empirical Bayes moderated t-Tests were applied, as implemented in the R/Bioconductor limma package (http://bioconductor.org/packages/release/bioc/html/limma.html). The resulting per protein and condition comparison p-values were adjusted for multiple testing using the Benjamini-Hochberg method.

##### Targeted LC-MS analysis of Neurexin immunoprecipitates and Exon 25 standards

Parallel reaction-monitoring (PRM) assays (Peterson et al., 2012) were generated from a mixture containing 50 fmol of each proteotypic heavy reference peptide (Table S5; JPT Peptide Technologies GmbH). To each peptide sample an aliquot of a heavy reference peptide mix containing chemically synthesized proteotypic peptides (Spike-Tides, JPT, Berlin, Germany) was spiked into each sample at a concentration of 2 fmol of heavy reference peptides per 1μg of total endogenous protein mass.

Targeted proteomic assays were validated by titration of recombinant proteins. Recombinant control proteins containing or lacking splice insertions (Nrxn3α-AS3(+)-AS4(+/-)-AS6(+)) were expressed in HEK293 cells as reported (Nguyen et al., 2016). A DNA construct for bacterial expression of a NRXN3 C-terminal fragment containing exons 25a, 25b, and 25c fused to GFP in the pET29b(+) backbone was generated using standard molecular biology procedures. The resulting GFP-fusion protein was purified from *E.coli* (BL21 strain) using Talon Metal Affinity Resin (Clontech) and eluted in 50mM NaH_2_PO_4_, 300mM NaCl, 250mM imidazole, 6M Urea.

Samples were subjected to LC–MS/MS analysis using a Orbitrap Fusion Lumos Mass Spectrometer fitted with an EASY-nLC 1200 (both Thermo Fisher Scientific) and a custom-made column heater set to 60°C. Peptides were resolved using a RP-HPLC column (75μm × 36cm) packed in-house with C18 resin (ReproSil-Pur C18–AQ, 1.9 μm resin; Dr. Maisch GmbH) at a flow rate of 0.2 μLmin-1. The following gradient was used for peptide separation: from 5% B to 50% B over 60 min o 95% B over 2 min followed by 18 min at 95% B. Buffer A was 0.1% formic acid in water and buffer B was 80% acetonitrile, 0.1% formic acid in water.

The mass spectrometer was operated in DDA mode with a cycle time of 3 seconds between master scans. Each master scan was acquired in the Orbitrap at a resolution of 120,000 FWHM (at 200 m/z) and a scan range from 300 to 1600 m/z followed by MS2 scans of the most intense precursors in the orbitrap at 30,000 FWHM (at 200 m/z) resolution with isolation width of the quadrupole set to 1.4 m/z. Maximum ion injection time was set to 50ms (MS1) and 50 ms (MS2) with an AGC target set to 1e6 and 1e5, respectively. Only peptides with charge state 2 – 5 were included in the analysis. Monoisotopic precursor selection (MIPS) was set to Peptide, and the Intensity Threshold was set to 5e3. Peptides were fragmented by HCD (Higher-energy collisional dissociation) with collision energy set to 35%, and one microscan was acquired for each spectrum. The dynamic exclusion duration was set to 30s.

The acquired raw-files were searched using the MaxQuant software (Version 1.6.2.3) against the same murine database mentioned above using default parameters except protein, peptide and site FDR were set to 1 and Lys8 and Arg10 were added as variable modifications. The best 6 transitions for each peptide were selected automatically using an in-house software tool and imported into SpectroDive (version 10, Biognosys, Schlieren). A unscheduled mass isolation list containing all peptide ion masses was exported form SpectroDive and imported into the Orbitrap Lumos operating software for PRM analysis.

Peptide samples for PRM analysis were resuspended in 0.1% aqueous formic acid, spiked with the heavy reference peptide mix at a concentration of 2 fmol of heavy reference peptides per 1 μg of total endogenous peptide mass and subjected to LC–MS/MS analysis on the same LC-MS system described above using the following settings: The resolution of the orbitrap was set to 30,000 FWHM (at 200 m/z), the fill time was set to 54 ms to reach an AGC target of 1e6, the normalized collision energy was set to 35%, ion isolation window was set to 0.4 m/z and the scan range was set to 150 – 1500 m/z. A MS1 scan at 120,000 resolution (at 200 m/z), AGC target 1e6 and fill time of 50 ms was included in each MS cycle. All raw-files were imported into SpectroDive for protein / peptide quantification. To control for variation in injected sample amounts, the total ion chromatogram (only comprising ions with two to five charges) of each sample was determined and used for normalization. To this end, the generated raw files were imported into the Progenesis QI software (Nonlinear Dynamics (Waters), Version 2.0), together with one standard shotgun analysis of a pooled sample using the same MS1 and gradient setting. Then, the intensity of all identified precursor ions with a charge of 2+ - 5+ were extracted, summed for each sample and used for normalization. Normalized ratios were transformed from the linear to the log-scale, normalized relative to the control condition and the median ratio among peptides corresponding to one protein was reported.

The mass spectrometry proteomics data have been deposited to the ProteomeXchange Consortium via the PRIDE (Perez-Riverol et al., 2022) partner repository with the dataset identifier PXD031379 and 10.6019/PXD031379 and to PanoramaWeb (https://panoramaweb.org/__r8753/project-begin.view).

#### Electrophysiology

##### Slice preparation for patch-clamp recordings

Electrophysiological experiments were performed using littermate male and female *Nrxn3*^*ΔEx24*^ +/+ and -/- mice aged between 6 and 8 weeks. Animals were anesthetized with isoflurane (4% in O_2_, Vapor, Draeger) and killed by decapitation. To increase cell viability, mice were exposed to oxygen-enriched atmosphere for 10 min before decapitation. Slices were obtained as previously described (Bischofberger et al., 2006; Heigele et al., 2016). Briefly, the brain was dissected in ice-cold sucrose-based solution (approximately 4°C) which was equilibrated with carbogen (95% O_2_/5% CO_2_). Transverse 350 μm thick hippocampal slices were cut with an approximate 20° angle to the dorsal surface using a VT1200 vibratome (Leica Microsystems). The sucrose-based solution for cutting and storage contained the following (in mM): 87 NaCl, 25 NaHCO_3_, 2.5 KCl, 1.25 NaH_2_PO_4_, 75 sucrose, 0.5 CaCl_2_, 7 MgCl_2_ and 10 glucose. Slices were incubated at 35°C for 30 min after cutting and subsequently stored at room temperature until experiments were performed.

##### Whole-cell voltage-clamp recordings

For electrophysiological recordings slices were transferred to a bath chamber and continuously perfused with oxygenated artificial cerebrospinal fluid (ACSF) containing (in mM): 125 NaCl, 25 NaHCO_3_, 25 glucose, 2.5 KCl, 1.25 NaH_2_PO_4_, 2 CaCl_2_, 1 MgCl_2_. The ACSF was equilibrated with carbogen (95% O_2_/5% CO_2_) at room temperature (21-24 °C), resulting in pH 7.4. Dentate gyrus granule cells were visualized using an AxioExaminer.D1 (Zeiss) and infrared differential interference contrast video microscopy. Patch pipettes were pulled from borosilicate glass tubing with a 2.0 mm outer diameter and 0.5 mm wall thickness (Hilgenberg) using a Flaming-Brown P-97 puller (Sutter Instruments). Patch pipettes had a resistance between 2.5 and 4 MΩ and were filled with an internal solution containing the following (in mM) for recordings of spontaneous and miniature postsynaptic currents and electrically evoked GABAergic and glutamatergic currents: 140 KCl, 10 EGTA, 10 HEPES, 2 MgCl_2_, 2 Na_2_ATP, 0.3 GTP, 1 phosphocreatine. The pH was adjusted to 7.3 with KOH. For recordings of optogenetically activated inputs from PV and SST interneurons internal solutions with the following compositions (all components in mM) were used: 125 K-gluconate, 20 KCl, 10 HEPES, 10 EGTA, 2 MgCl_2_, 2 Na_2_ATP, 0.3 GTP, 1 phosphocreatine (for PV inputs) and 120 Cs-gluconate, 20 CsCl, 10 HEPES, 10 EGTA, 2 MgCl_2_, 2 Na_2_ATP, 2 TEACl, 0.3 GTP, 1 phosphocreatine (for SST inputs). KOH and CsOH were used to adjust the pH to 7.3, respectively.

Recordings were obtained using a Multiclamp 700B amplifier (Molecular Devices), filtered at 10 kHz, and digitized at 20 kHz with a CED Power 1401 interface (Cambridge Electronic Design). Data acquisition was controlled using IGOR Pro 6.31 (Wave Metrics) and the CFS library support from Cambridge Electronic Design. Recordings were only included if the initial seal resistance was > 5 times higher than the input resistance of the cells typically ranging from 5-15 GΩ. In most recordings, series resistance (R_s_ = 10-25 MΩ) was compensated with a correction of 80%, and experiments were discarded if R_s_ changed by > 25%.

##### Extracellular synaptic stimulation

For electrical stimulation of synaptic inputs, pipettes (resistance: 5-7 MΩ) were filled with HEPES-buffered 3 M NaCl solution and used to apply brief negative current pulses (0.2 ms). To stimulate both proximal dendritic and perisomatic GABAergic synaptic inputs onto dentate gyrus granule cells, two stimulation pipettes were placed into the outer half of the inner molecular layer (IML) and into the granule cell layer (GCL) near the hilar border. The stimulation intensity in GCL was about 20 μA (22.5 ± 2.7 μA, *wild-type* versus 18.8 ± 1.4 μA, *Nrxn3*^*ΔEx24*^ mice, p=0.4580), very similar to the intensity in IML (24.5 ± 2.7 μA, *wild-type* versus 22.7 ± 1.8 μA, *Nrxn3*^*ΔEx24*^ mice, p=0.8017). Synapses were stimulated by either single stimuli or double pulses (inter-stimulus interval: 100 ms) to determine paired-pulse ratios for both stimulation sites. To identify possible crosstalk between stimulation electrodes (due to their physical proximity), GCL stimulation pulses and IML stimulation pulses were either applied independently or sequentially (inter-stimulus interval: 100 ms). Recordings were discarded if preceding GCL stimulation changed the amplitude of the IML-evoked postsynaptic current (PSC) by > 20%. To stimulate glutamatergic afferent perforant-path synapses, the stimulation electrode was placed into the molecular layer. Glutamatergic PSCs were evoked by single pulses with a stimulation intensity of 20 μA. Stimulation artifacts were truncated in example traces for clarity.

##### Channelrhodopsin-mediated activation of GABAergic interneurons

A diode laser (DL-473, Rapp Optoelectronic) was coupled to the epifluorescent port of the microscope (Zeiss Examiner, equipped with a 40X water immersion objective; Carl Zeiss Microscopy GmbH, Jena, Germany) via fiber optics. The field of illumination was targeted to the granule cell layer for stimulation of PV interneurons, and to the outer molecular layer for stimulation of SST interneurons. TTL-controlled light pulses of 2 ms duration and 1-7 mW intensity (a range within which saturation of the postsynaptic current amplitude was reached) were used to stimulate both PV and SST interneurons.

##### Data Analysis

Patch-clamp data was analyzed offline using the open source analysis software Stimfit (https://neurodroid.github.io/stimfit; (Guzman et al., 2014)) and customized scripts written in Python. Amplitudes, paired-pulse ratios and decay times of evoked GABAergic and glutamatergic PSCs were analysed using average traces of at least 5 repetitions. For the analysis of spontaneous GABAergic and glutamatergic PSCs a template-matching algorithm, implemented in Stimfit (Clements and Bekkers, 1997; Jonas et al., 1993), was used as described previously (Schmidt-Salzmann et al., 2014). Automatically detected events were visually controlled and false positive events were deleted. The remaining events were fitted with the sum of two exponential functions revealing the amplitude, rise time and decay time of the spontaneous PSCs. Standard electrophysiological parameters were determined using a negative voltage step of 5 mV. The input resistance was calculated measuring the plateau current in response to the voltage step, whereas the capacitance was determined by fitting a biexponential function to the capacitive current at the onset of the voltage step.

##### Pharmacology

Spontaneous and evoked GABAergic and glutamatergic PSCs were recorded in the presence of the following ionotropic receptor blockers: NBQX (10 μM) and D-AP5 (25 μM) for GABAergic currents and Picrotoxin (100 μM) for glutamatergic currents. Tetrodotoxin (TTX, 0.5-1 μM) was used to block AP firing during miniature recordings. All drugs were stored as aliquots at -20°C and diluted in ACSF within a maximum of 2 days prior to recording. NBQX (20 mM, Tocris Bioscience, 1044) was dissolved in DMSO. D-AP5 (50 mM; Tocris Bioscience, 0106) and TTX (1 mM; Alomone Labs, T-550) was dissolved in water. Picrotoxin (50 mM; Sigma-Aldrich, P1675) was dissolved in ethanol.

### Quantification and statistical analysis

Statistical analysis was conducted with GraphPad Prism 7. Sample sizes were chosen based on previous experiments and literature surveys. No statistical methods were used to pre-determine sample sizes. Exclusion criteria used throughout this manuscript were pre-defined. There are detailed descriptions in the respective sections of the methods. Group assignment was defined by genotype, thus, no randomization was necessary. During initial pilot experiments, investigators were not blinded to genotype during data collection and/or analysis. Subsequent acquisition and analysis of larger datasets for quantification of synaptic markers and electrophysiological recordings were done by an investigator blinded to genotype. Appropriate statistical tests were chosen based on sample size and are indicated in individual experiments.

## Data Availability

The mass spectrometry proteomics data have been deposited to the ProteomeXchange Consortium via the PRIDE (Perez-Riverol et al., 2022) partner repository with the dataset identifier ProteomeXchange: PXD031379 and 10.6019/PXD031379 and to PanoramaWeb (https://panoramaweb.org/__r8753/project-begin.view). This paper does not report original code. Any additional information required to reanalyze the data reported in this paper is available from the lead contact upon request.

## Supplemental information

Document S1. Figures S1–S7

Table S1. List of proteins recovered in anti-NRXN3-AS5 immunoprecipitates, related to Figure 4 iBAQ values for proteins recovered in anti-HA immunoprecipitates from hippocampal tissue lysates from *wild-type* and *Nrxn3 AS5*^*HA/HA*^ mice. Proteins enriched in the precipitates from *Nrxn3 AS5*^*HA/HA*^ mice (FC > 2.0, q-value <0.05) are marked in bold.

Table S2. List of proteins recovered in anti-NRXN1-PAN immunoprecipitates, related to Figure 4 iBAQ values of proteins recovered in anti-NRXN1 and control IgG immunoprecipitates from hippocampal tissue lysates of wild-type mice.

Table S3. List of known NRXN binding partners recovered in NRXN1 and NRXN3 immunoprecipitates, related to Figure 4 iBAQ values of protein abundance for a hand-curated list of known extracellular Neurexin ligands and their detection in anti-HA immunoprecipitates from hippocampal tissue lysates of *wild-type* and *NRXN3 AS5*^*HA/HA*^ mice or detection in anti-NRXN1 and control IgG immunoprecipitates from hippocampal tissue lysates of wild-type mice, respectively.

Table S4. Method supplement, related to STAR Methods Oligonucleotide primer used for flanking primer PCRs or qPCR assays and 3’UTR sequences which were cloned into dual-promotor luciferase plasmids.

Table S5. List of stable isotope-labeled “heavy” peptides used for targeted proteomic assays, related to STAR Methods Note that some NRXN3 proteoform are not annotated within the “canonical” Uniprot database entries. In such cases the multiple Uniprot IDs are provided with marking of the canonical forms in red.

Table S6. Targeted proteomics *Nrxn3*^*ΔEx24*^ mice data supplement, related to Figure 5 All “light to heavy” ratios from targeted proteomics assays of NRXN1,2,3 on cortical, cerebellar and hippocampal tissue.

Document S2. Article plus supplemental information
