# Supplementary material for: Targeted proteoform mapping uncovers specific Neurexin-3 variants required for dendritic inhibition
Source: Neuron. 2022 Jul 6;110(13):2094–2109.e10. doi: 10.1016/j.neuron.2022.04.017 (PMC9275415; doi:10.1016/j.neuron.2022.04.017)
Supplement: Document S1. Figures S1–S7 [file mmc1.pdf]

Neuron, Volume *110*

Supplemental information

Targeted proteoform mapping uncovers specific Neurexin-3 variants required for dendritic inhibition

David Hauser, Katharina Behr, Kohtarou Konno, Dietmar Schreiner, Alexander Schmidt, Masahiko Watanabe, Josef Bischofberger, and Peter Scheiffele

Figure S1

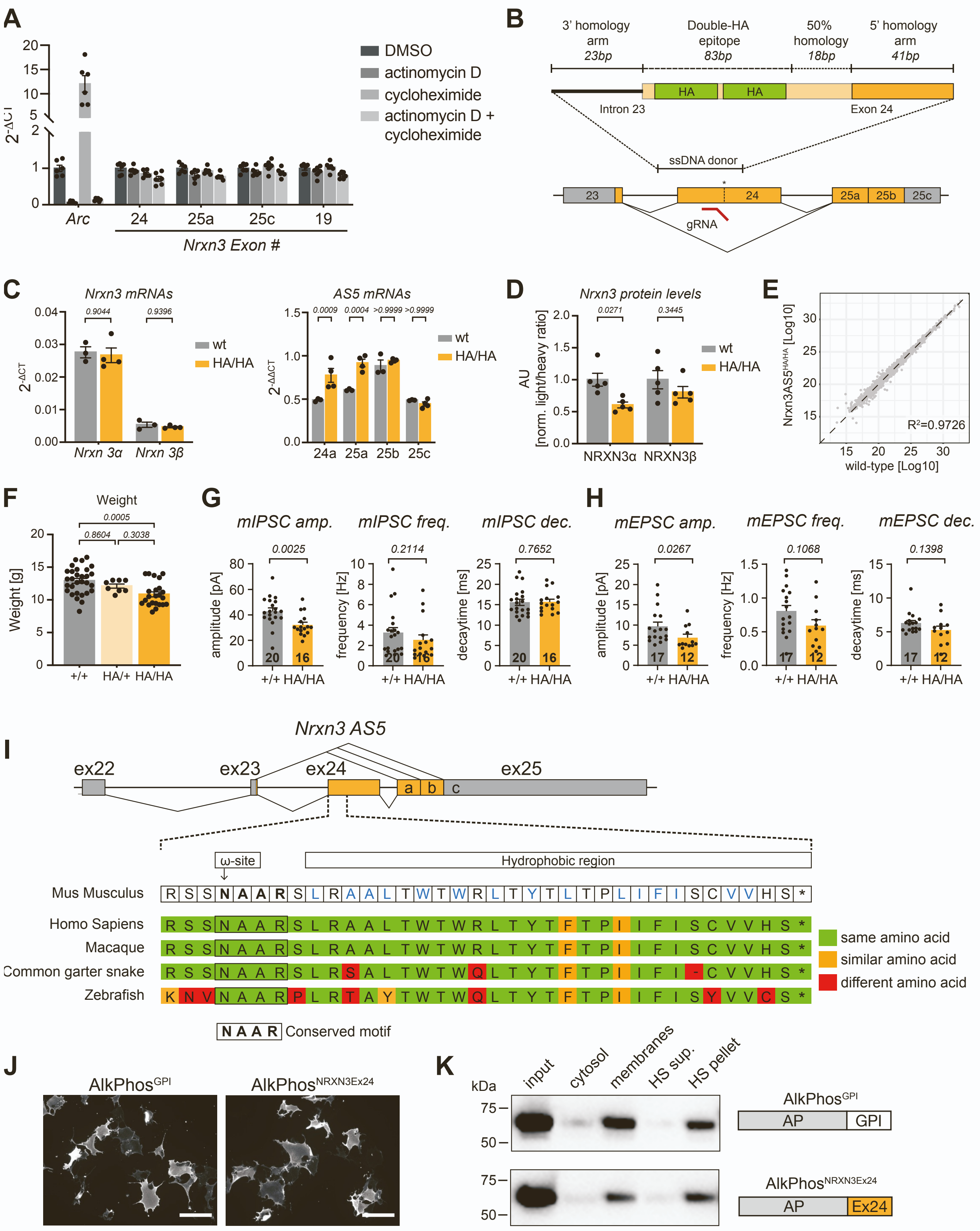

A
DMSO
actinomycin D
cycloheximide
actinomycin D + cycloheximide
Arc
24
25a
25c
19
Nrxn3 Exon #
B
3' homology arm 23bp
Double-HA epitope 83bp
50% homology 18bp
5' homology arm 41bp
HA
HA
Intron 23
Exon 24
ssDNA donor
23
24
25a
25b
25c
gRNA
C
Nrxn3 mRNAs
0.9044
0.9396
wt
HA/HA
Nrxn 3α
Nrxn 3β
AS5 mRNAs
0.0009
0.0004
>0.9999
>0.9999
24a
25a
25b
25c
D
Nrxn3 protein levels
0.0271
0.3445
AU [norm. light/heavy ratio]
NRXN3α
NRXN3β
E
R²=0.9726
wild-type [Log10]
F
Weight
0.0005
0.8604
0.3038
Weight [g]
+/+
HA/+
HA/HA
G
mIPSC amp.
mIPSC freq.
mIPSC dec.
0.0025
0.2114
0.7652
amplitude [pA]
frequency [Hz]
decaytime [ms]
20
16
+/+ HA/HA
H
mEPSC amp.
mEPSC freq.
mEPSC dec.
0.0267
0.1068
0.1398
17
12
I
Nrxn3 AS5
ex22
ex23
ex24
ex25
a
b
c
ω-site
Hydrophobic region
Mus Musculus
R S S N A A R S L R A A L T W T W R L T Y T L T P L I F I S C V V H S *
Homo Sapiens
R S S N A A R S L R A A L T W T W R L T Y T F T P I I F I S C V V H S *
Macaque
R S S N A A R S L R A A L T W T W R L T Y T F T P I I F I S C V V H S *
Common garter snake
R S S N A A R S L R S A L T W T W Q L T Y T F T P I I F I - C V V H S *
Zebrafish
K N V N A A R P L R T A Y T W T W Q L T Y T F T P I I F I S Y V V C S *
same amino acid
similar amino acid
different amino acid
N A A R Conserved motif
J
AlkPhos^GPI
AlkPhos^NRXN3Ex24
K
input
cytosol
membranes
HS sup.
HS pellet
kDa
75
50
AlkPhos^GPI
AP
GPI
AlkPhos^NRXN3Ex24
AP
Ex24

Figure S1. Validation of GPI-anchored Neurexin 3 expression (addition to Figure 1)

(A) Quantitative RT-PCR analysis of mRNAs from cortical cultures treated with actinomycin D (10µg/ml), cycloheximide (25µg/ml) or both and DMSO only control, normalized to *Gapdh*, N=2 cortical culture preparations, n=3 culture well replicates per preparation, DIV12, *Arc* = Activity-regulated cytoskeleton-associated protein.

(B) Schematic of HA epitope-tag insertion into exon 24 of *Nrxn3*. AS5 with alternative donor- and acceptor splice sites or alternative exon (yellow) and constitutive exon (grey) indicated below with position of gRNA recognition site indicated, single stranded DNA donor and homology regions indicated on top, with homology arms and insert labeled.

(C) Quantitative PCR analysis of hippocampal RNA from *wild-type* and homozygous *Nrxn3AS5*$^{HA/HA}$ knock-in mice, for both major *Nrxn3* (left panel, normalized to *Gapdh*) and AS5 splice isoforms mRNAs (right panel, normalized to *Gapdh and PAN-Nrxn3*), N=3-4 mice per genotype.

(D) Protein level assessment of *wild-type* and homozygous *Nrxn3AS5*$^{HA/HA}$ knock-in mice by targeted proteomics. Ratios of light to heavy peptide detection are displayed in reference to *wild-type* samples as average values for 2 heavy peptides. Note that NRXN3 beta expression in the hippocampus is very low resulting in higher variability of measurements. The reduction in NRXN3 protein output is likely due to translational silencing through RNA elements characterized in Figure 5.   Hippocampus, P25-30, N=5 mice per genotype.

(E) Shotgun proteomics displayed as relative MS abundance comparing overall proteome of *wild-type* and homozygous *Nrxn3AS5*$^{HA/HA}$ knock-in mice. Hippocampus, P25-30, N=5 mice per genotype.

(F) Weight of *wild-type* and heterozygous or homozygous *Nrxn3AS5*^{HA} littermates, P25-30, N=8-30 mice.

(G) Amplitude, frequency and decay time constants of miniature inhibitory currents of *wild-type* and homozygous *Nrxn3AS5*^{HA} mice, P42-56, N=3 mice per genotype.

(H) Amplitude, frequency and decay time constants of miniature excitatory currents of wild-type and homozygous *Nrxn3AS5*^{HA} mice, P42-56, N=3 mice per genotype.

(I) Schematic representation of AS5 of *Nrxn3* and evolutionary conservation between species of the coding region of exon 24. Identical amino acids are highlighted green, amino acids with

similar side chains in orange, divergent amino acids in red, large hydrophobic amino acids in the mouse sequence are marked in blue.

(J) Immunostainings of COS7-cells with overexpressed HA-tagged alkaline phosphatase constructs with endogenous GPI-anchoring sequence (left) or fused in frame to the exon 24 coding sequence of *Nrxn3* (right).

(K) Western Blot of subcellular fractionation of cytosolic, membrane, and high-salt (HS) washed membrane fractions (equal percentage of total sample loaded in all lanes) from transfected HEK293 cells expressing HA-tagged alkaline phosphatase constructs.

Mean and SEM, two-way ANOVA followed by Bonferroni’s test (C,D, and F) or Mann-Whitney test (G and H), Pearson correlation (E). Scale bar in (J) is 100 µm.

Figure S2

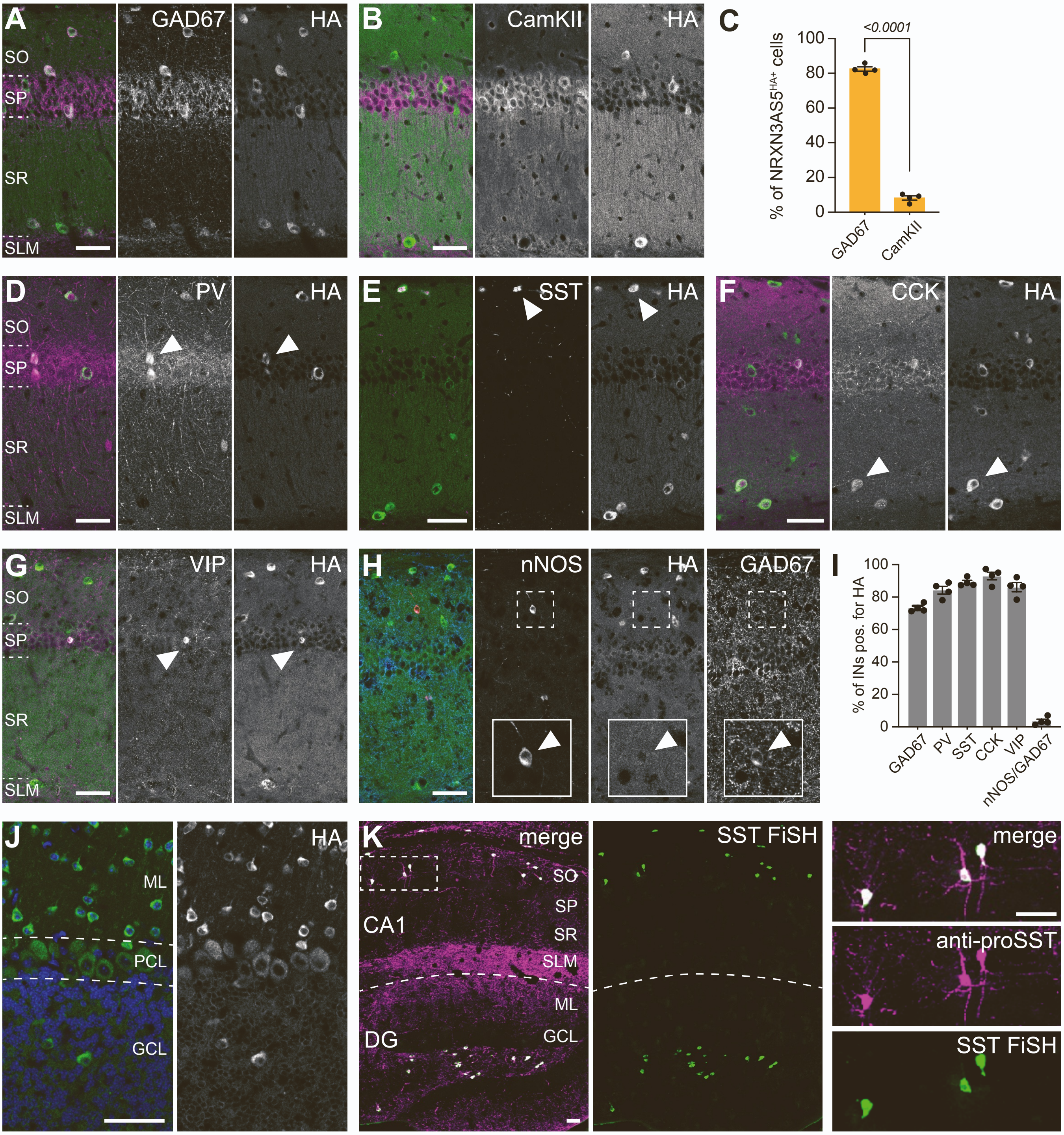

A
GAD67
HA
SO
SP
SR
SLM
B
CamKII
HA
C
% of NRXN3AS5HA+ cells
<0.0001
100
80
60
40
20
0
GAD67
CamKII
D
PV
HA
E
SST
HA
F
CCK
HA
G
VIP
HA
H
nNOS
HA
GAD67
I
% of INs pos. for HA
PV
SST
CCK
VIP
nNOS/GAD67
J
HA
ML
PCL
GCL
K
merge
SST FiSH
CA1
DG
anti-proSST

Figure S2. Selective expression of NRXN3 AS5+ proteoforms (addition to Figure 2)

(A,B) Co-expression analysis of NRXN3 $AS5^{HA}$ protein (HA, green) and GAD67 or CamKII (magenta) in CA1, SO = stratum oriens, SP = stratum pyramidale, SR = stratum radiatum, SLM = stratum lacunosum moleculare.

(C) Fraction of NRXN3 $AS5^{HA}$ positive cells co-expressing GAD67 or CamKII, N=4 mice, n=3-4 brain slices per mouse, P25-30.

(D-H) Co-expression analysis of NRXN3 $AS5^{HA}$ protein (HA, green) with interneuron markers (magenta) parvalbumin (PV), somatostatin (SST), cholecystokinin (CCK), VIP and nNOS/GAD67 in CA1. Arrowheads show example co-labeled cells. Inset in H shows magnified soma of cell.

(I) Fraction of GABAergic interneurons in CA1 co-expressing NRXN3-$AS5^{HA}$, N=4 mice, n=3-4 brain slices per mouse, P25-30. Note that for nNOS only cells also positive for GAD67 were counted.

(J) Immunochemical detection of HA-tagged proteins in cerebellum from homozygous *Nrxn3-AS5^HA/HA^* knock-in mice (HA, green, DAPI, blue), ML = molecular layer, PCL = purkinje cell layer, GCL = granule cell layer.

(K) Validation of newly generated anti-pro-SST antibody (magenta) showing co-labeling of SST mRNA-expressing cells (detected by fluorescent in situ hybridization, green) in mouse hippocampus.

Mean and SEM, students t-test, scale bar is 50 µm.

Figure S3

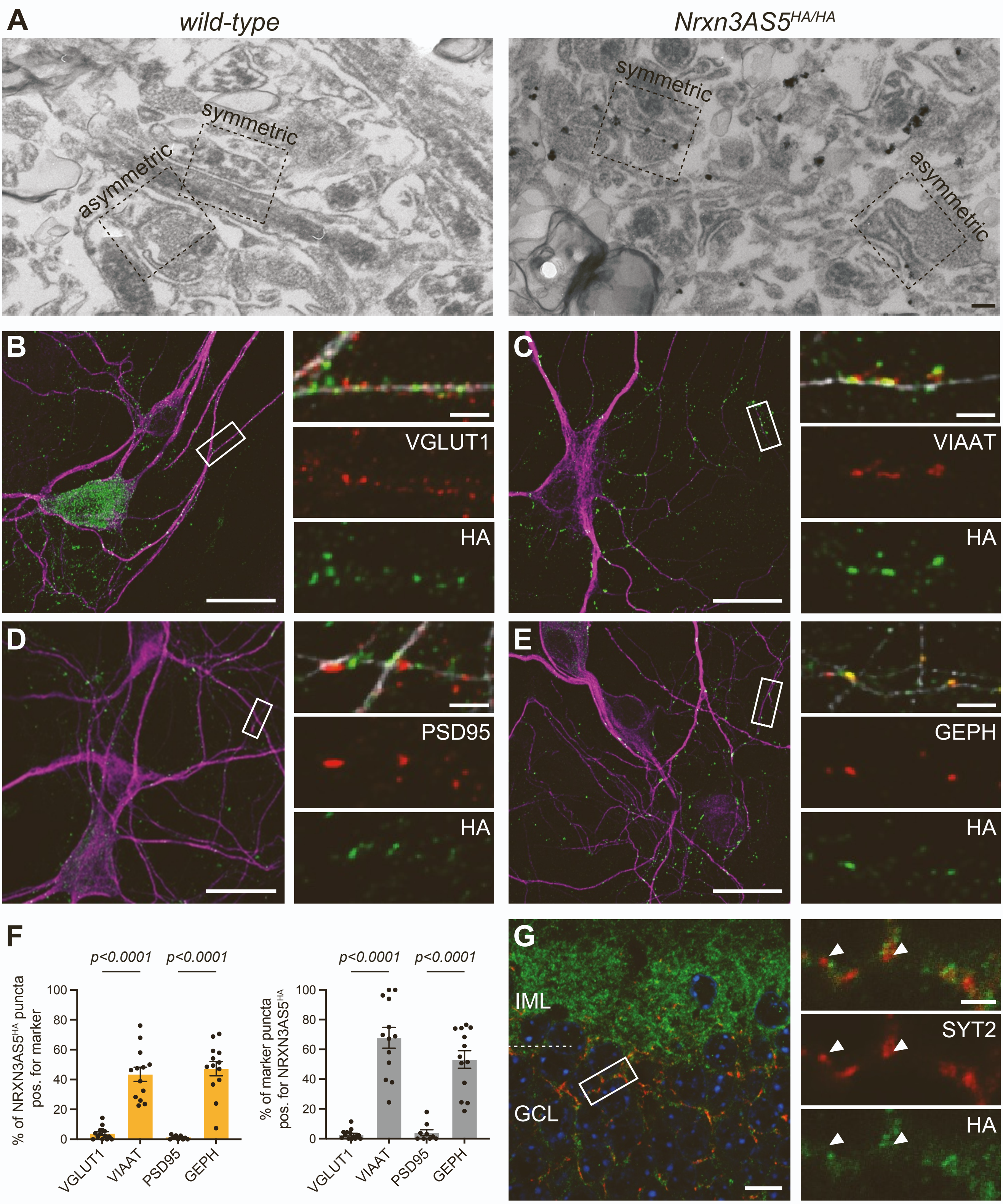

A
wild-type
Nrxn3AS5$^{HA/HA}$
symmetric
asymmetric
symmetric
asymmetric
B
VGLUT1
HA
C
VIAAT
HA
D
PSD95
HA
E
GEPH
HA
F
p<0.0001
p<0.0001
% of NRXN3AS5^{HA} puncta pos. for marker
100
80
60
40
20
0
VGLUT1
VIAAT
PSD95
GEPH
p<0.0001
p<0.0001
% of marker puncta pos. for NRXN3AS5^{HA}
100
80
60
40
20
0
VGLUT1
VIAAT
PSD95
GEPH
G
IML
GCL
SYT2
HA

Figure S3. Synaptic expression of NRXN3 AS5+ proteoforms (addition to Figure 3)

(A) Overview images of pre-embedding immunoelectron microscopy shown in Figure 2D.

(B-E) Immunocytochemistry of day in vitro 10 hippocampal cultures from *Nrxn3AS5*$^{HA/HA}$ knock-in mice with co-labeling of pre-synaptic markers vGlut1 (B) or VIAAT (C) and post-synaptic markers PSD95 (D) or Gephyrin (E) (MAP2, magenta/white, HA, green, pre- and post-synaptic markers, red). Note that the labeled soma in panel B is a GABAergic cell whereas all other somata shown are glutamatergic.

(F) Quantification of synaptic localization displaying the percentage of HA-positive puncta co-labeled with pre- and post-synaptic markers (left) and percentage of respective pre- and post-synaptic markers co-labeled with anti-HA immunostaining (right). Mean and SEM, one-way ANOVA followed by Bonferroni's test, N=2 hippocampal cultures, n=6-8 ROI (one per coverslip) per condition, 60-100 puncta per ROI.

(G) High magnification view of NRXN3-AS5^{HA} protein (HA, green) and synaptotagmin 2 (SYT2, red) localization in mouse dentate gyrus (P28), IML = inner molecular layer, GCL = granule cell layer. Arrowheads indicate SYT2 positive puncta negative for HA labeling.

Scale bar is 200 nm in (A), 20 μm in (B-E), 10 μm in (G) and 2 μm in the enlarged regions of interest in (B-E) and (G).

Figure S4

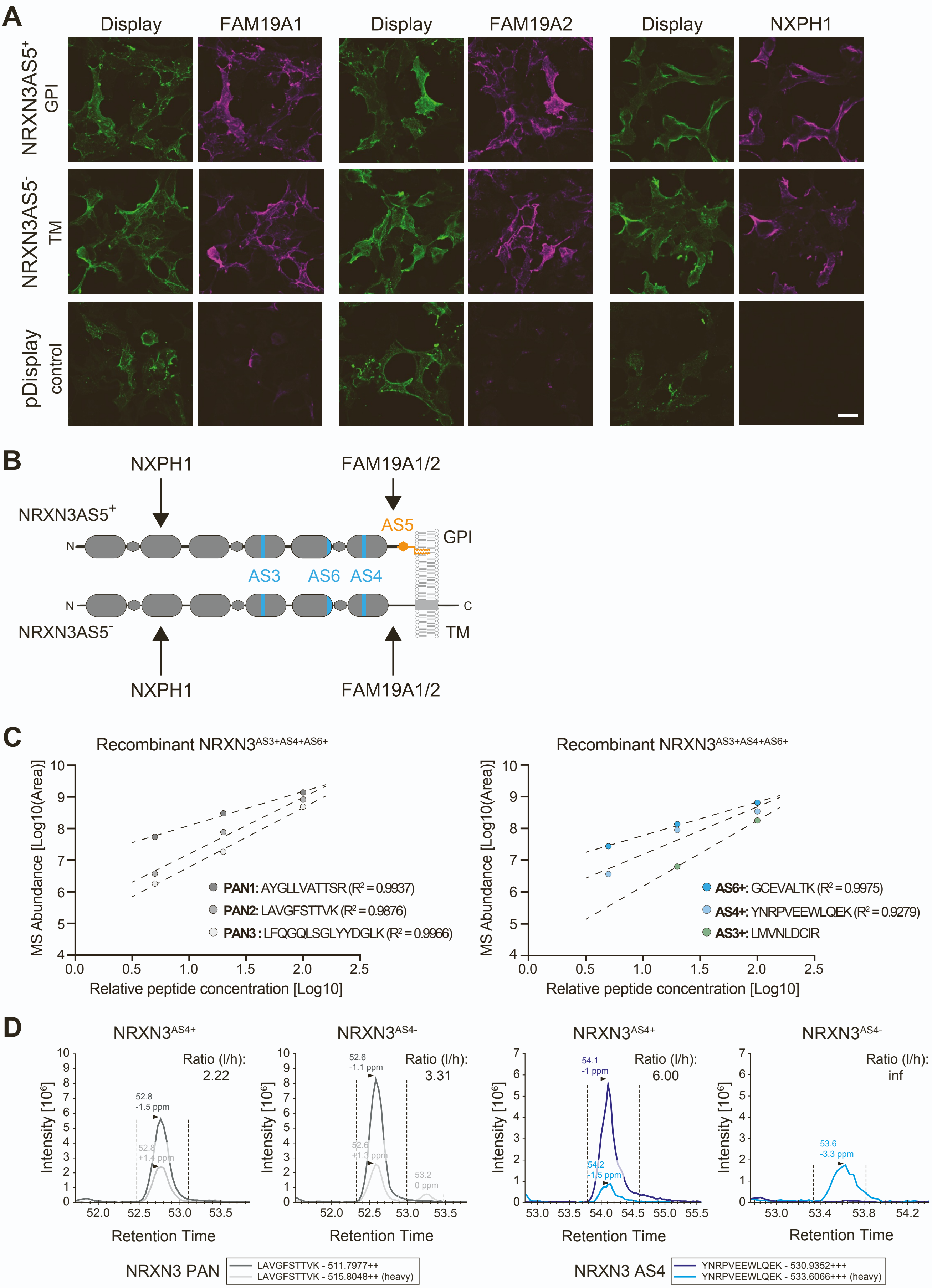

A
Display
FAM19A1
Display
FAM19A2
Display
NXPH1
NRXN3AS5+ GPI
NRXN3AS5- TM
pDisplay control
B
NXPH1
FAM19A1/2
NRXN3AS5+
AS5
GPI
N
AS3
AS6
AS4
C
NRXN3AS5-
TM
C
Recombinant NRXN3AS3+AS4+AS6+
MS Abundance [Log10(Area)]
Relative peptide concentration [Log10]
PAN1: AYGLLVATTSR (R² = 0.9937)
PAN2: LAVGFSTTVK (R² = 0.9876)
PAN3 : LFQGQLSGLYYDGLK (R² = 0.9966)
AS6+: GCEVALTK (R² = 0.9975)
AS4+: YNRPVEEWLQEK (R² = 0.9279)
AS3+: LMVNLDCIR
D
NRXN3AS4+
NRXN3AS4-
Ratio (l/h): 2.22
Ratio (l/h): 3.31
Ratio (l/h): 6.00
Ratio (l/h): inf
Intensity [10⁶]
Retention Time
52.8 -1.5 ppm
52.8 +1.4 ppm
52.6 -1.1 ppm
52.6 +1.3 ppm
53.2 0 ppm
54.1 -1 ppm
54.2 -1.5 ppm
53.6 -3.3 ppm
NRXN3 PAN
LAVGFSTTVK - 511.7977++
LAVGFSTTVK - 515.8048++ (heavy)
NRXN3 AS4
YNRPVEEWLQEK - 530.9352+++
YNRPVEEWLQEK - 533.6066+++ (heavy)

Figure S4. Binding partner and targeted proteomic assay validation (addition to Figure 4)

(A) Co-expression and cell surface labeling of HEK293T cells co-transfected with GPI-anchored NRXN3 AS5+, transmembrane NRXN3 AS5-, or pDisplay (driving membrane-bound CFP), together with NXPH1, FAM19A1, and FAM19A2, respectively. Both NRXN3 constructs had the following splice insertions: AS1+, AS2-, AS3+, AS4+, AS6+.

(B) Illustration of position of NXPH1 and FAM19A1/2 interaction sites in the NRXN3AS5+ and AS5- ectodomains.

(C) Assay development for detection of NRXN3 PAN peptides (left) and peptides detecting alternative splice insertions at AS3, AS4, AS6 (right). Titration of increasing amounts of recombinant NRXN3 protein expressed in HEK293 cells. Note that the MS abundance for NRXN3 PAN peptides in anti-HA immunoprecipitates plotted in Figure 3 is 7.5-9.0, i.e. well within the sensitivity range covered by this assay.

(D) Endogenous and heavy reference peptide traces for NRXN3 PAN and NRXN3 AS4 peptides in samples containing recombinant NRXN3 protein with and without splice insertion at AS4.

Scale bar in (A) is 20μm.

Figure S5

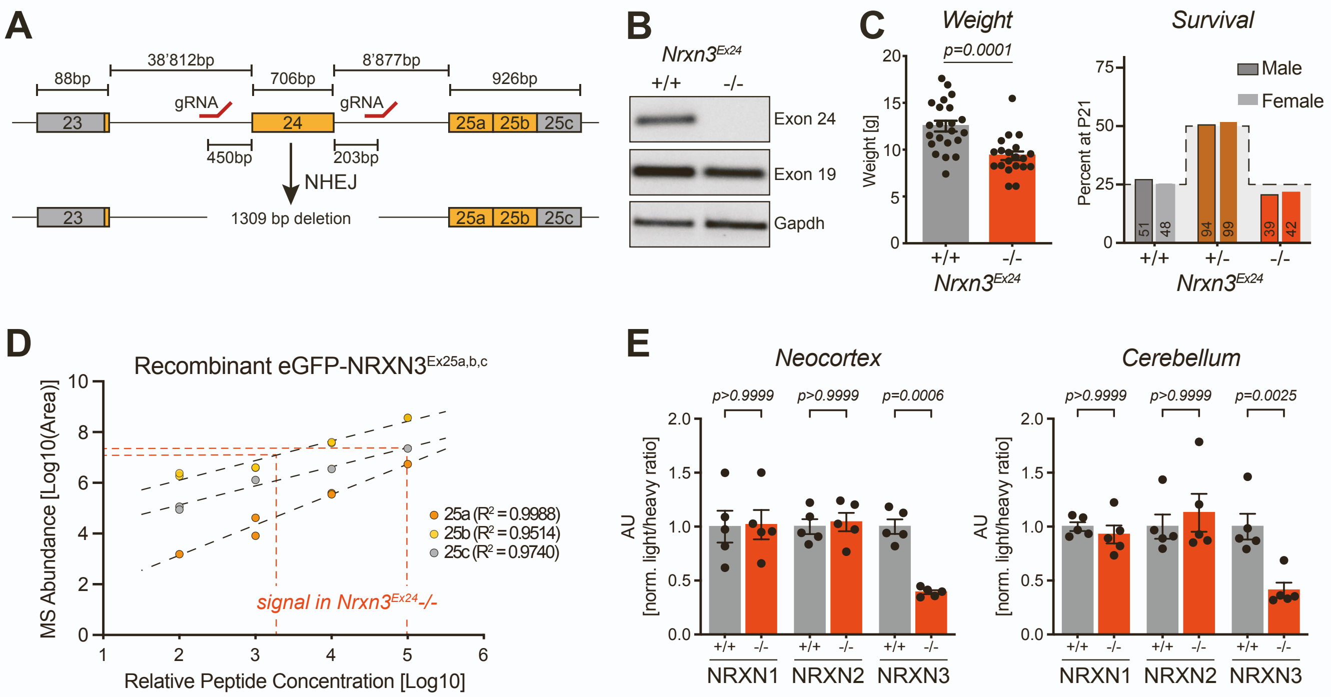
A
88bp
38'812bp
706bp
8'877bp
926bp
gRNA
gRNA
23
24
25a 25b 25c
450bp
203bp
NHEJ
1309 bp deletion
B
Nrxn3Ex24
+/+ -/-
Exon 24
Exon 19
Gapdh
C
Weight
p=0.0001
Weight [g]
Nrxn3Ex24
Survival
Male
Female
Percent at P21
D
Recombinant eGFP-NRXN3Ex25a,b,c
MS Abundance [Log10(Area)]
Relative Peptide Concentration [Log10]
25a (R² = 0.9988)
25b (R² = 0.9514)
25c (R² = 0.9740)
signal in Nrxn3Ex24-/-
E
Neocortex
Cerebellum
p>0.9999
p=0.0006
p=0.0025
AU [norm. light/heavy ratio]
NRXN1 NRXN2 NRXN3

Figure S5. Properties of *Nrxn3^{ΔEx24}* mice (addition to Figure 5)

(A) Schematic diagram illustrating the generation of *Nrxn3^{ΔEx24}* mice by Crispr/Cas9 gene editing with two gRNAs targeting introns upstream and downstream of exon 24. Sizes of exons (boxes) and introns (dashed lines), and position of gRNA recognition sites are indicated. Non-homologous end-joining (NHEJ) resulted in a 1309 bp deletion.

(B) Semi-quantitative PCR amplifying *Nrxn3* transcript regions containing alternative exon 24 or the constitutive exon 19 or *Gapdh* control from mouse hippocampus.

(C) Weight of *wild-type* and *Nrxn3^{ΔEx24}* littermates, P25-30, N=20-21 mice (left panel). The cause for the weight reduction is unknown but reduced weight is also reported for global deletion of all NRXN3 isoforms. Survival of *wild-type*, heterozygous and homozygous *Nrxn3^{ΔEx24}* mice in relation to expected Mendelian ratios for male and female mice at P21, total animal numbers are indicated within columns (right panel).

(D) Assay development for detection of peptides encoded by alternative acceptor sites 25a and 25b and constitutive exon 25c. Titration of a recombinant protein containing all three alternative segments probed with PRM assays for 25a, 25b and 25c. Signal level of endogenous peptides for 25b and 25c from *Nrxn3^{ΔEx24}* hippocampus indicated with dashed lines (average of N=5 mice).

(E) Detection of NRXN1, NRXN2, NRXN3 by targeted proteomics with heavy peptides targeting proteotypic peptides shared by all NRXN1, NRXN2 and NRXN3 proteoforms. Ratios of light to heavy peptide detection are displayed in reference to *wild-type* samples of each peptide individually, one representative peptide shown for cerebellum and cortical samples, consistent results were obtained for multiple proteotypic peptides for the same proteoform (See Table S6), P25-30, N=5 mice per genotype and brain area.

Mean and SEM, student’s t-test (C) or two-way ANOVA followed by Bonferroni’s test (E).

Figure S6

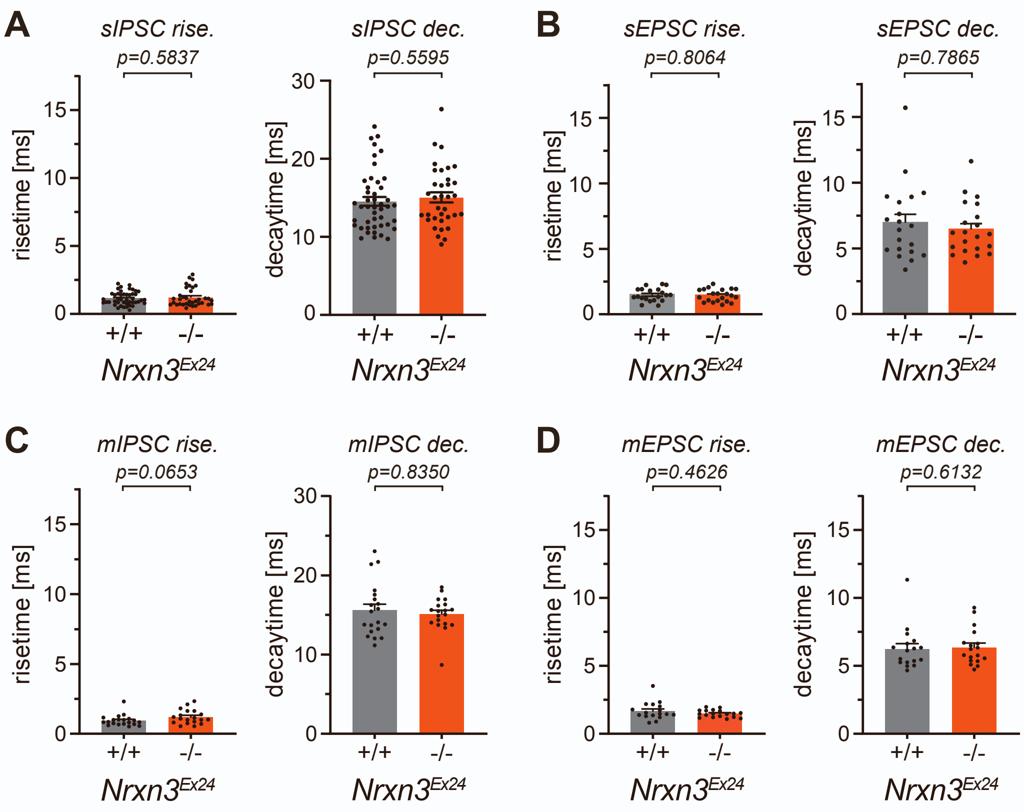
A
sIPSC rise.
p=0.5837
risetime [ms]
sIPSC dec.
p=0.5595
decaytime [ms]
B
sEPSC rise.
p=0.8064
risetime [ms]
sEPSC dec.
p=0.7865
decaytime [ms]
C
mIPSC rise.
p=0.0653
risetime [ms]
mIPSC dec.
p=0.8350
decaytime [ms]
D
mEPSC rise.
p=0.4626
risetime [ms]
mEPSC dec.
p=0.6132
decaytime [ms]
+/+
-/-
Nrxn3Ex24

Figure S6. Kinetics of spontaneous events and miniature postsynaptic currents in *Nrxn3*$^{\Delta Ex24}$ mice (addition to Figure 6)

(A) Kinetics (rise and decay time) of dentate gyrus granule cell sIPSCs recorded from *wild-type* (N=16 animals, n=45 cells) and homozygous *Nrxn3*$^{\Delta Ex24}$ mice (N=12 animals, n=35 cells).

(B) Kinetics (rise and decay time) of dentate gyrus granule cell sEPSCs recorded from *wild-type* (N=4 animals, n=20 cells) and homozygous *Nrxn3*$^{\Delta Ex24}$ mice (N=4 animals, n=21 cells).

(C) Kinetics (rise and decay time) of dentate gyrus granule cell mIPSCs recorded from *wild-type* (N=3 animals, n=20 cells) and homozygous *Nrxn3*$^{\Delta Ex24}$ mice (N=3 animals, n=19 cells).

(D) Kinetics (rise and decay time) of dentate gyrus granule cell mEPSCs recorded from *wild-type* (N=3 animals, n=17 cells) and homozygous *Nrxn3*$^{\Delta Ex24}$ mice (N=3 animals, n=18 cells).

Mean and SEM, Mann-Whitney test.

Figure S7

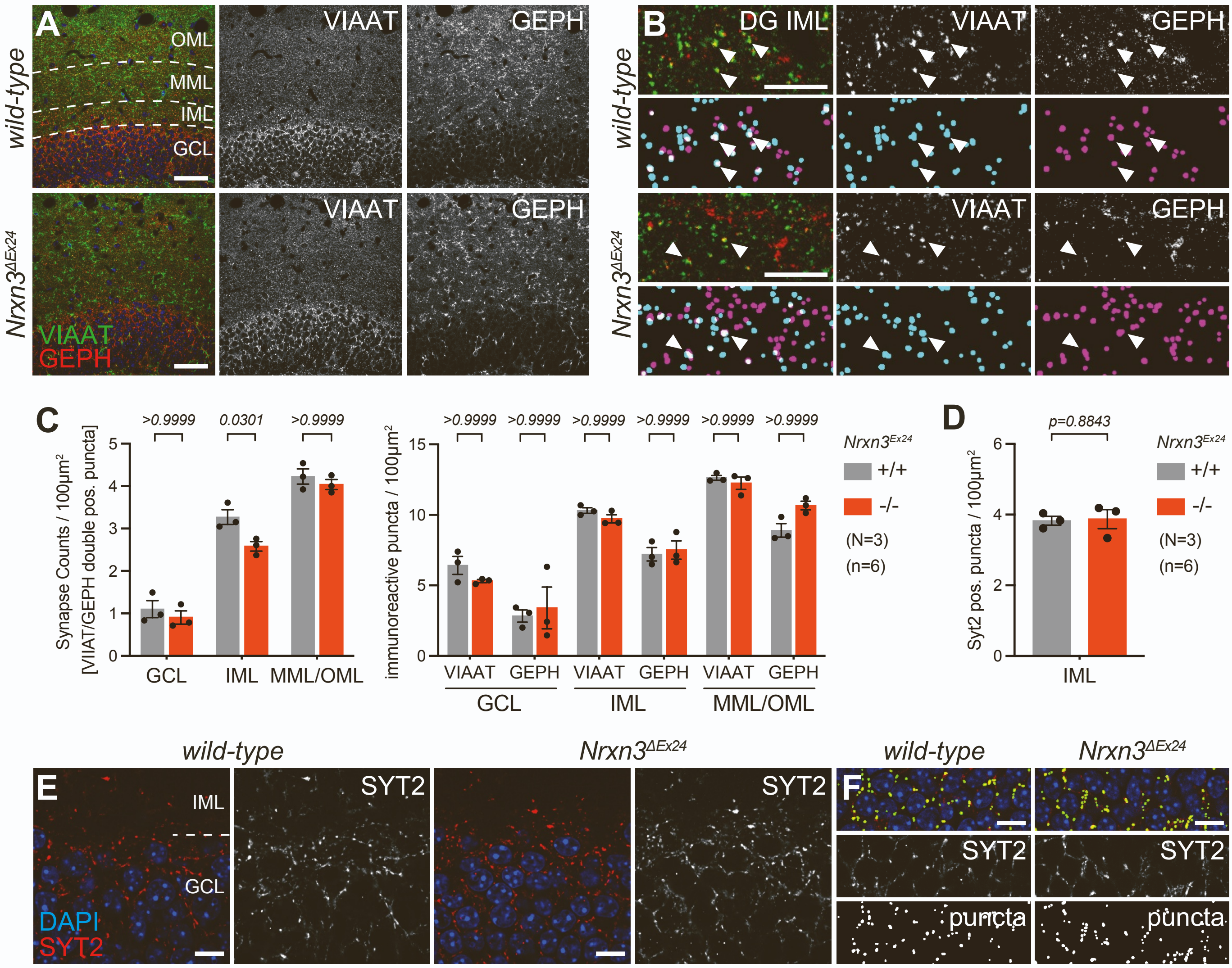

A
wild-type
OML
MML
IML
GCL
VIAAT
GEPH
Nrxn3ΔEx24
B
DG IML
C
>0.9999
0.0301
Synapse Counts / 100µm²
[VIIAT/GEPH double pos. puncta]
GCL
IML
MML/OML
immunoreactive puncta / 100µm²
Nrxn3Ex24
+/+
-/-
(N=3)
(n=6)
D
p=0.8843
Syt2 pos. puncta / 100µm²
E
F
DAPI
SYT2
puncta

Figure S7. Synapse number analysis of *Nrxn3*$^{\Delta Ex24}$ mice (addition to Figure 7)

(A) Immunochemical detection of synaptic markers VIAAT (green) and Gephyrin (red) in dentate gyrus of hippocampus from *wild-type* mice (top panel) and *Nrxn3*$^{\Delta Ex24}$ mice (bottom panel), OML = outer molecular layer, MML = middle molecular layer, IML = inner molecular layer, GCL = granule cell layer.

(B) High magnification view of synaptic markers VIAAT (green) and Gephyrin (red) in dentate gyrus IML from *wild-type* mice (top panels) and *Nrxn3*$^{\Delta Ex24}$ mice (bottom panels). Positive puncta for VIAAT (cyan) and Gephyrin (magenta) are shown as detected by the synapse counter plug-in and example overlapping puncta (white) are indicated by arrowheads.

(C) Quantification of immunoreactive puncta for VIAAT/Gephyrin double-positive structures (left panel) and for pre- (VIAAT) and post-synaptic (Gephyrin) markers (right panel) in granule cell (GCL), inner molecular (IML) and middle/outer molecular (MML/OML) layers of *wild-type* and *Nrxn3*$^{\Delta Ex24}$ mice (N=3 animals per genotype, n=6 brain slices per animal).

(D) Quantification of immunoreactive puncta for Synaptotagmin 2 (SYT2) in dentate gyrus inner molecular layer (IML) of *wild-type* and *Nrxn3*$^{\Delta Ex24}$ mice (N=3 animals per genotype, n=6 brain slices per animal).

(E) Synaptotagmin 2 (SYT2, red) expression in dentate gyrus of *wild-type* and *Nrxn3*$^{\Delta Ex24}$ mice, IML = inner molecular layer, GCL = granule cell layer.

(F) High magnification view of Synaptotagmin 2 (SYT2, red) and detected puncta of SYT2 (green) in dentate gyrus GCL of *wild-type* and *Nrxn3*$^{\Delta Ex24}$ mice.

Mean and SEM, two-way ANOVA followed by Bonferroni's test (C) or student's t-test (D). Scale bar is 50 µm in (A), 10 µm in (B,E, and F).
